# LANDMark: an ensemble approach to the supervised selection of biomarkers in high-throughput sequencing data

**DOI:** 10.1186/s12859-022-04631-z

**Published:** 2022-03-31

**Authors:** Josip Rudar, Teresita M. Porter, Michael Wright, G. Brian Golding, Mehrdad Hajibabaei

**Affiliations:** 1 Department of Integrative Biology & Centre for Biodiversity Genomics, University of Guelph, 50 Stone Road East, Guelph, ON N1G 2W1 Canada; 2 Department of Biology, McMaster University, 1280 Main St. West, Hamilton, ON L8S 4K1 Canada

**Keywords:** Biomarker selection, Metagenomics, Metabarcoding, Biomonitoring, Ecological assessment, Machine learning

## Abstract

**Background:**

Identification of biomarkers, which are measurable characteristics of biological datasets, can be challenging. Although amplicon sequence variants (ASVs) can be considered potential biomarkers, identifying important ASVs in high-throughput sequencing datasets is challenging. Noise, algorithmic failures to account for specific distributional properties, and feature interactions can complicate the discovery of ASV biomarkers. In addition, these issues can impact the replicability of various models and elevate false-discovery rates. Contemporary machine learning approaches can be leveraged to address these issues. Ensembles of decision trees are particularly effective at classifying the types of data commonly generated in high-throughput sequencing (HTS) studies due to their robustness when the number of features in the training data is orders of magnitude larger than the number of samples. In addition, when combined with appropriate model introspection algorithms, machine learning algorithms can also be used to discover and select potential biomarkers. However, the construction of these models could introduce various biases which potentially obfuscate feature discovery.

**Results:**

We developed a decision tree ensemble, LANDMark, which uses oblique and non-linear cuts at each node. In synthetic and toy tests LANDMark consistently ranked as the best classifier and often outperformed the Random Forest classifier. When trained on the full metabarcoding dataset obtained from Canada’s Wood Buffalo National Park, LANDMark was able to create highly predictive models and achieved an overall balanced accuracy score of 0.96 ± 0.06. The use of recursive feature elimination did not impact LANDMark’s generalization performance and, when trained on data from the BE amplicon, it was able to outperform the Linear Support Vector Machine, Logistic Regression models, and Stochastic Gradient Descent models (*p* ≤ 0.05). Finally, LANDMark distinguishes itself due to its ability to learn smoother non-linear decision boundaries.

**Conclusions:**

Our work introduces LANDMark, a meta-classifier which blends the characteristics of several machine learning models into a decision tree and ensemble learning framework. To our knowledge, this is the first study to apply this type of ensemble approach to amplicon sequencing data and we have shown that analyzing these datasets using LANDMark can produce highly predictive and consistent models.

**Supplementary Information:**

The online version contains supplementary material available at 10.1186/s12859-022-04631-z.

## Background

Biomarkers represent a broad category of objectively measurable characteristics which can identify, or at least provide evidence for, a particular biological process or biological entity such as an organism or specific tissue/cell type. Amplicon sequence variants (ASVs), which are unique variations of specific DNA sequences, can be considered a class of biomarker [1]. These sequences are typically generated by the amplification and sequencing of a specific region of a marker gene, such as cytochrome oxidase I, and are used to study patterns in biodiversity [1]. The complex and noisy nature of high-throughput sequencing (HTS) data sets presents a significant hurdle in identifying important ASV biomarkers. Statistical and machine learning models can fail to identify important co-variates (features in machine learning) due to underlying problems within the data set and the assumptions of each analytical method [2–7]. For example, the detection of novel patterns, effect of higher order interactions, and non-linear relationships between taxa are not effectively evaluated using linear statistical models [8–10]. Noise and/or incorrect modelling due to various distributional assumptions also contributes to difficulties in development and use of predictive models. This can then present itself as a hurdle when using these models to identify important predictive attributes [11–13]. The efficiency of the amplification step, the amount of error introduced during amplification and sequencing, the sampling process, and the storage of samples represent some of the entry points through which noise is introduced noise into HTS datasets [14]. Bioinformatic methods that attempt to account and correct for noise can also lead to differences in results. For example, different denoising algorithms and distance metrics could have a substantial impact when measuring alpha and beta diversity [15]. Finally, it is important to note that many older marker-gene sequencing studies made use of clusters of sequencing reads, referred to as operational taxonomic units (OTUs), which differ by less than an arbitrary dissimilarity threshold [1]. Since ASVs are not sets of similar sequences, the number of ASVs can be considerably larger than the number of OTUs. This can be a problem since the use of ASVs increases the dimensionality of the final data sets. This can make it difficult to find potential biomarkers using statistical models since the number of multiple comparisons which are performed could mask potentially important results [16, 17].

Two possible, but not mutually exclusive, avenues can be taken to find potential biomarkers in HTS data. These are the algorithmic (predictive) and statistical modelling approaches [7]. While a detailed discussion of these approaches and their applicability to metabarcoding research is outside the scope of this paper, the merits of these approaches have been and continue to be extensively debated and explored in the literature for at least two decades [7]. On one hand, algorithmic modelling is concerned with using the collected data to arrive at sensible and accurate predictions [18]. However, the interpretation of these models is often difficult, computationally expensive, and not intuitive [19, 20]. Statistical models, on the other hand, attempt to find a particular statistical distribution which best fits the data. Therefore, a model is built assuming that the data follows the underlying assumptions of the statistical distribution and allow for an easy understanding of how each feature is associated with the outcome of interest [7, 10]. In statistical models, however, bias and sources of error could be introduced due to the assumptions about the data and choices made before applying the model to the data [10, 11]. Despite extensive debates, neither method is clearly superior but rather complementary. For example, a statistical framework can be used to test for differences in predictive performance between models [21, 22]. Thus, the choice of approach should depend on the goals of the study and the characteristics of the data. Finally, these methods can also be used to validate the findings of the other [23].

Unfortunately, real data sets do not usually obey one or more distributional assumptions. For example, MetagenomeSeq is an R package designed to look for differentially abundant sequences and assumes that sequencing counts follow a zero-inflated log normal distribution [24]. LEfSe (Linear Discriminant Analysis Effect Size) is a tool that uses abundance information to identify biomarkers and assumes multivariate normality [11, 25]. Assumptions about the proportion of differentially abundant ASVs also occur [11]. Layered over this is the fact that amplicon sequencing datasets are compositional in nature [26, 27]. This necessitates the need to develop or explore more suitable models for indicator or differential abundance problems [11, 28]. Therefore, to identify potential biomarkers, models which are capable of handling sparseness, compositionality, and limiting the impact of noise are preferable.

Investigations into the ability of statistical models to identify biomarkers has shown that different methods may not consistently detect the same biomarkers, likely due to differing assumptions about the underlying distribution of each ASV’s counts [29, 30]. As a result, it is often difficult to choose a particular statistical model. Machine learning approaches represent a powerful alternative way to analyze and interpret data. However, the effectiveness of these approaches in modelling marker-gene data is under-explored and under-utilized [31]. While it is true that these models are often complex, recent innovations in understanding how features are used to arrive at predictions are making these approaches far more accessible [19, 20, 32]. Machine learning approaches are also attractive since they tend to be considered as a class of non-parametric models and therefore assume very little or nothing about the underlying data. In fact, their goal is to approximate a decision boundary which best separates outcomes. The quality of this approximation is then measured as the ability to predict outcomes on unseen or new data and/or to infer which factors can be used for predictive purposes. For example, recent work has shown that eDNA metabarcoding data can be used to infer biotic indices without depending on a reference database [33]. While these approaches are powerful and are being increasingly used in ecological research, just like statistical models, they must be used with care. This is because biases used in the construction of an accurate predictive model may result in the inability of the model to discover a good approximation of the underlying data generation function.

This work introduces the Large-Scale Non-parametric Discovery of Markers (LANDMark) method. LANDMark attempts to discover an accurate separation between samples by building an ensemble of decision trees capable of making oblique cuts. Traditionally, decision trees used in Random Forests and Extremely Randomized Trees make axis-aligned cuts at each node. Despite the strengths of these methods, the decision boundaries learned by these methods can be highly problematic, especially when these models are trained on data containing highly correlated features [13]. Oblique and non-linear cuts—which use the information from multiple features to discriminate between classes—is one way to avoid this issue [13, 34]. LANDMark considers both types of splits at each node to construct decision trees which are better able to explore the geometry of the underlying data in a simple and elegant manner while retaining many of the useful properties of decision trees [9, 34, 34, 35]. We will investigate LANDMark’s generalization performance using toy, synthetic, and real data while contrasting our approach against other commonly used machine learning models. Finally, we will measure the ability of our algorithm to select a consistent set of ASVs associated with outcomes of interest. The significance of these features will be examined in the context of the broader literature.

## Methods

### Toy datasets

Toy data can provide valuable insight into how various algorithms perform across a wide variety of domains. Since they are well studied, these datasets provide a sanity check ensuring that new data analysis algorithms function in line with what is reported in the literature. Five of the datasets (Parkinson’s Speech, Seeds, Heart Failure, Raisin, and Coimbra Breast Cancer) were downloaded from the UCI Machine Learning Repository [36–43]. The Iris, Wisconsin Breast Cancer, and Wine datasets were obtained from the Scikit-Learn library [44]. The concentric circles’ and ‘two moons” datasets were constructed using Scikit-Learn while the ‘two spirals’ dataset was constructed using code obtained from [45]. Counts of OTUs from the v3–5 region of the bacterial 16S rRNA gene were downloaded from https://www.hmpdacc.org/HMQCP/ [46]. Samples corresponding to the tongue dorsum, saliva, and sub-gingival plaque were then extracted and used for a subsequent analysis. Detailed descriptions of each dataset, any normalization, and their construction (in the case of the ‘concentric circles’, ‘two spirals’, and ‘two moons’ datasets) can be found in Additional file 1: Table S4.

### Amplicon sequencing dataset selection

We selected a previously published real-world dataset for testing. The dataset was derived from benthic tissue samples but collected from wetlands in Wood Buffalo National Park in Alberta, Canada using two COI amplicons, F230 and BE [47–50]. This dataset was chosen since it represents a diverse boreal wetland, the dataset can be divided into two smaller datasets characterized by non-overlapping amplicons, and it has been extensively studied [50–54]. These properties make this dataset a suitable choice for testing a new biomarker discovery algorithm. The outcomes of interest in each of these datasets are ASVs can be used to identify the different wetland habitats (Peace River vs Athabasca River).

### Bioinformatics

Raw sequences in the FASTQ format were obtained from the Hajibabaei Lab at the University of Guelph [47, 52]. Demultiplexed paired-end Illumina reads were then processed using a COI metabarcode pipeline available from https://github.com/Hajibabaei-Lab/SCVUC_COI_metabarcode_pipeline [55]. Cytochrome Oxidase I (COI) samples were first paired using SeqPrep with a minimum Phred score of 20 in the overlap region [56]. Primers and adapter sequences were removed using CutAdapt 1.10 and only fragments of at least 150 bp were kept [57]. This stage also removed any sequences with more than 3 ambiguous nucleotides and a Phred score of less than 20 at either end of the fragment. The remaining quality controlled sequences were extracted and de-replicated with VSEARCH 2.8.2 [58]. The unoise3 functionality of USEARCH 10.0.240 was then used to denoise (remove globally rare clusters and chimeric reads) the de-replicated set of reads into ASVs [6, 59]. VSEARCH 2.8.2 was then used to construct the ASV count matrix and the RDP Classifier (version 2.12) was used to assign taxonomy to the COI reads using a curated database of COI sequences (COI Classifier v3.2) [55, 60].

Following the initial processing steps, ASVs with a bootstrap support of 0.3 or higher at the genus level were chosen for further analysis. These cut-offs were chosen to reduce the probability of a false taxonomic assignment for COI amplicons ~ 200 bp in length with the assumption that the query sequence is represented in the reference sequence database [55, 60]. Only ASVs belonging to taxa which could confidently be assigned to macroinvertebrates used in biomonitoring were selected [61]. Data sets were constructed such that each amplicon could be analyzed independently. Raw counts were stored in a site by ASV count matrix. Unless otherwise specified, each raw sample by ASV matrix was converted into presence-absence and filtered so only ASVs found in two or more samples are retained. This step was taken since the goal of this study is to identify ASVs which are shared between samples of the same origin and that reducing the size of the feature space can often lead to a more generalizable model [26, 29, 62]. Furthermore, presence-absence transformation can introduce some biases due to weighting rare and common taxa equally. However, unlike rarefaction, this transformation retains ASV occurrence information. Rarefaction and centered log-ratio transformations (CLR) were not used due to the potential biases that may be introduced when taking read abundance into account. For example, in very sparse datasets the CLR transformation begins to approach normalizing by total sum scaling if there are many absences. Rarefaction is problematic since it will omit random subsets of ASVs and introduce unwanted variation into the data [12, 26]. Processed amplicon sequencing data and indicator species results can be found in Additional file 6.

### The LANDMark algorithm

**Fig. 1:**
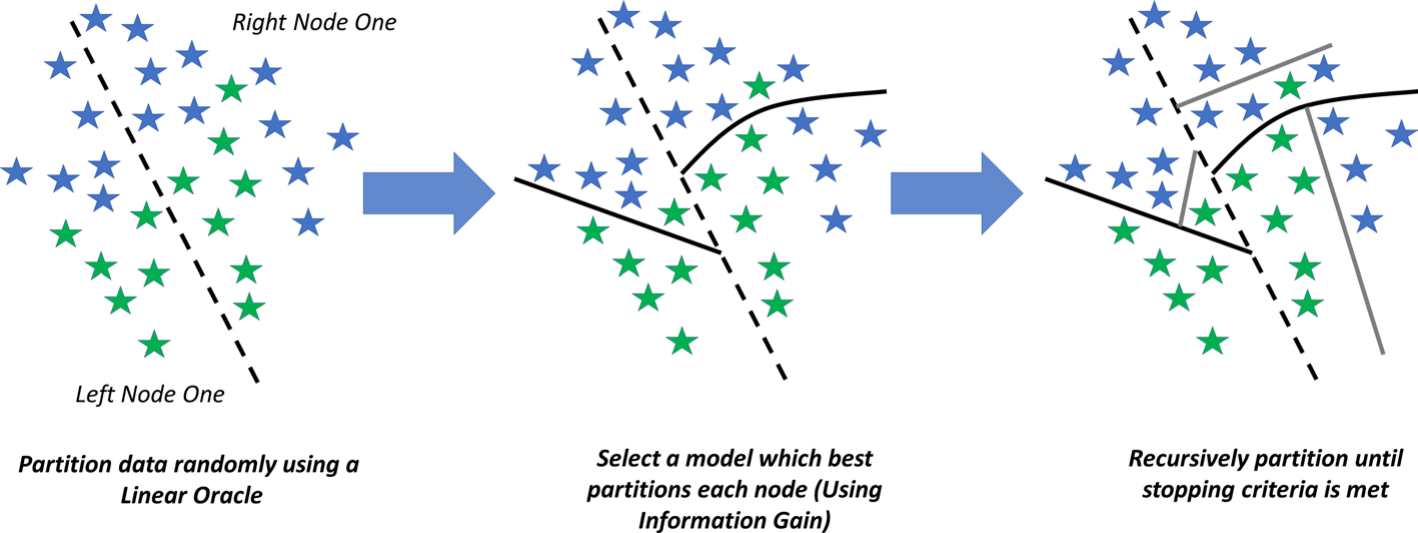
**A geometric overview of how LANDMark trees can partition samples.** Oblique (straight line) and non-linear (curved line) splits are created linear and neural network models, respectively. Unlike the axis aligned splits used in Random Forests, LANDMark nodes consider multiple features. This can allow each model to take advantage of the additional information and use it to learn more appropriate decision rules. Since multiple models are considered at each node, only those which partition samples into smaller purer regions are selected. Random linear oracles, left, can be used to add additional randomness to LANDMark. This approach selects two points at random without replacement and calculates the midpoint between these samples. This midpoint is then used to find the hypersurface orthogonal to the initial two points. Samples are then partitioned according to side of the hypersurface in which they are found. Following this, different randomized subsets of features and/or a bootstrapped samples of the data are then used to train different supervised models in each node (middle). This process is repeated until a stopping criterion is met (right). Many trees are constructed in this way and their decisions combined to produce the final prediction

**Fig. 2:**
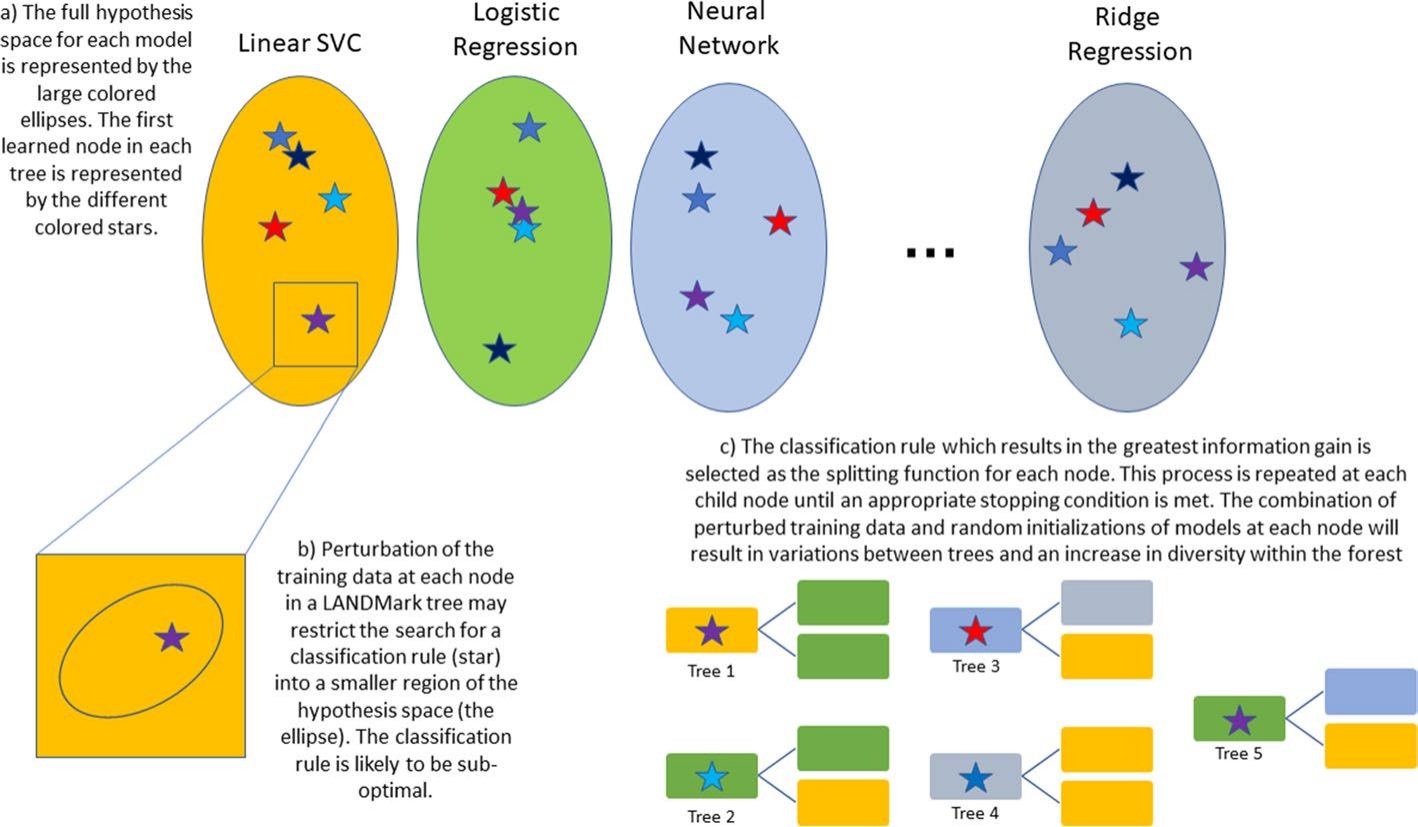
**A simplified overview of how LANDMark constructs and selects splitting rules at each node.** Multiple models are evaluated at each node in each tree. Diversity between LANDMark trees is due to the random selection of training samples and features in each node, random initializations of most models, and the random selection of models which create partitions with equivalent information gain

The overall algorithm for LANDMark is presented in Additional file 5. Briefly, a LANDMark decision tree will select either an oblique or non-linear split at each node. Oblique and non-linear splits use information from multiple features to discover a hyperplane which best models training data. The hyperplane is simply a decision rule that is not necessarily parallel to the feature axis. Similarly, non-linear splits use information from multiple features to learn a plane-curve which best models the training data. LANDMark accomplishes this by training multiple classification models at each node using a perturbed dataset. A simplified geometric overview of this is presented in Fig. 1. Ultimately, each node will learn a set of classification rules about the original data and only rules which result in the greatest information gain, which are more likely to reflect the underlying geometric structure of the data, are selected as splitting functions (Fig. 2) [34]. We believe that our approach to constructing decision trees will result in the evaluation of a diverse set of splitting functions. Additional randomness, which is known to improve performance of decision tree models, could also be injected into the model by way of a random linear oracle [35].The final goal of this approach will hopefully result in an improvement of each tree’s approximation of the decision boundary. Taken together as an ensemble, the improvements to each tree can then be combined by averaging to create a more accurate classification model [8, 13, 63]. The source code can be found at: https://github.com/jrudar/LANDMark.

The Scikit-Learn library is used by LANDMark to train linear models at each LANDMark node. LANDMark will train a logistic regression, ridge regression, linear support vector machine (LSV), and stochastic gradient descent (SGD) model at each node [44]. Each linear model has a hyperparameter which controls the regularization strength of the model. If more than 6 samples enter the node, a cross-validated grid search is conducted to find an appropriate regularization strength for the model. In the case of the SGD classifiers, the grid search is used to select an appropriate loss function. Settings for each model’s hyperparameters can be found in Table 1.

**Table 1:** Default models and the possible parameter choices for each model under different conditions

Classifier	Non-tunable parameters	Number of training features (≥ 4 features)	Parameters (If number of samples > 6)	Parameters (if number of samples ≤ 6)
Logistic regression (LBFGS solver)	max_iter = 2000 penalty = “l2”	Randomly selected—user defined	A grid search using fivefold stratified cross-validation is used to choose C from logarithmically spaced values in the range of 10^–4^ and 10^4^	C parameter set to 1.0
Logistic regression (Liblinear solver)	max_iter = 2000 penalty = “l1”	Full	A grid search using fivefold stratified cross-validation is used to choose C from logarithmically spaced values in the range of 10^–4^ and 10^4^	C parameter set to 1.0
Linear SVC	max_iter = 2000	Randomly selected—user defined	A grid search using fivefold stratified cross-validation is used to choose alpha (for SGD classifiers) or C (for the linear SVC). The possible choices for these parameters are 0.001, 0.01, 0.1, 1.0, 10, 100. In the case of the SGD Classifier, the loss function (hinge or modified Huber) is also chosen using 5 cross-validation	C parameter set to 1.0
Stochastic gradient descent classifier (L2 penalty)	max_iter = 2000	Randomly selected—user defined	A grid search using fivefold stratified cross-validation is used to choose alpha (for SGD classifiers) or C (for the linear SVC). The possible choices for these parameters are 0.001, 0.01, 0.1, 1.0, 10, 100. In the case of the SGD Classifier, the loss function (hinge or modified Huber) is also chosen using 5 cross-validation	Alpha parameter set to 1.0, loss function (hinge or modified Huber) is randomly chosen
Stochastic gradient descent classifier (elastic-net penalty)	max_iter = 2000	Full	A grid search using fivefold stratified cross-validation is used to choose alpha (for SGD classifiers) or C (for the linear SVC). The possible choices for these parameters are 0.001, 0.01, 0.1, 1.0, 10, 100. In the case of the SGD Classifier, the loss function (hinge or modified Huber) is also chosen using 5 cross-validation	Alpha parameter set to 1.0, loss function (hinge or modified Huber) is randomly chosen
Ridge regression	NA	Randomly selected—user defined	Alpha chosen from logarithmically spaced values in the range of 10^–3^ and 10^4^ using generalized cross validation	NA
Neural network	batch_size = 32 epochs = 300 validation_split = 0.10 min_delta = 0.0001 patience = 40 See text for architecture details	Randomly selected—user defined	NA	NA

LANDMark can also explore possible non-linear decision boundaries using a neural network model created using Tensorflow [64]. The architecture of this model is described below and an overview is presented in Additional file 4: Fig. S12 while Table 1 provides an overview of each model used by LANDMark and the possible parameters which may be used. The neural network used by LANDMark is only used if there are more than 32 samples entering the node. This choice was deliberate since training this network is computationally expensive. The network used by LANDMark is split into two smaller networks, each using the same sample by ASV matrix as input data. The first network contains five hidden layers with 256, 128, 64, 48, and 24 hidden units in each hidden layer. Alpha dropout, with a dropout rate of 0.2, is used after the first, second, and third hidden layers. This form of dropout prevents overfitting by randomly setting a fraction of each layer’s activations to their negative saturation value [65]. This encourages the network to learn a more general representation of the input data. The activation function of each hidden layer is the “mish” function [66]. This function is a non-linear and helps the network learn how to represent non-linearities which could be present in the input data. Input data is also passed into a Random Fourier Features layer. This layer projects the original data into a 24-dimensional representation [67]. These features then pass through three hidden layers with 24, 24, and 16 nodes. The output of each sub-network is then concatenated, and concatenated layer is connected to a softmax layer where the number of hidden units is equal to the number of classes. For example, in the Wood Buffalo training data there are two classes which correspond to the Athabasca and the Peace River Deltas. Categorical cross-entropy, the loss function for the network, is optimized using an Adam optimizer with a learning rate of 0.001) [68]. The data used to train the network is randomly divided into 90% training data and 10% validation data. The network runs for a maximum of 300 epochs unless the loss on the validation data is at or below 10^–4^ for 40 epochs.

### Model choices, parameterizations, and hardware

LANDMark was compared to the following classifiers: Extremely Randomized Trees (ET), SGD Classifier using the squared hinge and modified Huber loss functions, Random Forest Classifier (RF), LSV, Logistic Regression CV, and Ridge Regression CV [44]. Models were run using their default settings. This was done since many prospective users will not deviate from these settings [69]. However, an exception was made for regularization parameters because two models, the Ridge and Logistic Regression classifiers attempt to select an appropriate parameter using cross-validation. A cross-validated grid search was used to select the regularization parameters for the LSV and SGD models. The best parameter was chosen from a list of logarithmically spaced values between 1e−4 and 1e4. All tests were run on a computer using an Intel Core i5-10400F CPU, 16 GB of RAM, and an NVIDIA GeForce GTX 1650 Super GPU with 4 GB of memory.

### Generation of synthetic datasets

The ability of LANDMark to identify predictive features, such as ASVs which are indicative of a particular condition, is important in validating the approaches presented in this paper. Synthetic data was constructed using the filtered data sampled in 2012 from the Athabasca River in Wood Buffalo National Park [47, 52]. The subset of the data containing the F230R amplicon was used to ensure a relative level of homogeneity within samples. However, the choice to use this subset was arbitrary. To simulate the effect of a treatment, a copy of the original samples was made and likelihood of the presence of an ASV was modified. We also created datasets which randomly perturbed 25, 50, or 75 ASVs to investigate how well models perform when the number of predictive ASVs differed. The likelihood for each selected ASV to be present in the treatment group was selected at random from the following list: 0.1, 0.125, 0.25, 0.5, 2, 4, 8, 10 and calculated by solving Eq. 1. In this equation, *p*_*o*_ is the original probability, *p*_*N*_ is the increase or decrease in likelihood, and *q* represents the new probability. The columns associated with these ASVs in the treatment set were then replaced with a new set of values constructed using the selected likelihoods. Additional randomness was also added by shuffling the presence and absences of 5% of ASVs. Each synthetic dataset contained 48 samples, 24 of which were controls. Thirty different datasets were created for each scenario.

1 $$log\frac{q}{1-q}=log\frac{{p}_{O}}{1-{p}_{O}}+log{p}_{N}$$

### Assessment of generalization performance using models trained with synthetic and toy datasets

To test for significant differences in generalization performance thirty different train-test splits, with the classes in each set being proportional to those in the original, were created for each toy data set. 80% of the original data was used to construct each training set. For reproducibility, the random state used to create each train-test split was set to the iteration number for the split. A model was trained using each training set and the balanced accuracy score (Eq. 2) calculated using the corresponding test set [70]. Balanced accuracy scores are calculated by first constructing a confusion matrix and then dividing the number of correct predictions for each treatment,${c}_{ii}$, by the greater of either its row or column sum, $\sum {c}_{i,}$, $\sum {c}_{,i}$. The results for each treatment are then summed and divided by the total number of treatments $n$ [70]. For synthetic data, each dataset was split into a training set, which contained 80% of the samples, and a test set which contained the remaining 20%.

Depending on the type of synthetic dataset the indices of the top 25, 50, or 75 features from each model were extracted and used to calculate the number of true positive (TP), false positive (FP), false negative (FN), and true negative (TN) ASVs. These values were then used to calculate the Mathews Correlation Coefficient (MCC) (Eq. 3), which can be used as a proxy for the false-discovery rate since it summarizes how successful each classifier is at identifying true positive features while minimizing the detection of false negatives [71]. The Friedman test, conducted using the ‘Real Statistics’ Excel package (Release 7.2) [72], was used to assess if there is any significant difference in the ability of each model to recognize real features. A Friedman test was also used to assess if a significant difference between the balanced accuracy scores could be detected [73]. If a significant result was found a Nemenyi post-hoc test was used to determine which pairs of models differed significantly.

We investigated the impact of three LANDMark hyperparameters on generalization performance: the number of estimators in the forest, the number of features considered, and the effect of the using the random oracle. Although LANDMark does make use of other hyperparameters, investigating these would be the most pertinent since they directly contribute to the diversity of ensemble. To investigate the effect of these parameters we constructed thirty synthetic dataset (as described above) with 75 features which differed between each dataset. Each dataset was divided into a training and testing dataset as described above. We then varied each parameter and measured the balanced accuracy score on the test data. LANDMark, RF, and ET classifiers were trained using 1, 2, 4, 8, 16, 32, 64, 128, and 256 trees. The ‘max_features’ parameter, which controls the number of features considered at each node, was tested at the following levels: the square root of the number of features, 0.1, 0.2, 0.3, 0.4, 0.5, 0.6, 0.7, and 0.8. Plots were created using the “relplot” function of the ‘seaborn’ package in Python 3.8. The 95% confidence interval, estimated using 10,000 bootstraps, was drawn around each parameter. We did not need to construct a synthetic dataset to measure the impact of the random oracle since this was tested as part of our generalization performance comparisons. Finally, we recognize that LANDMark is likely to be a computationally expensive algorithm since it trains a learned model at each node. To gauge the impact of this we created thirty different synthetic datasets using the ‘make_classification’ function from Scikit-Learn [44]. Each dataset contained 100 samples, and 500 features, 25 of which were informative. All other parameters were kept at their defaults. The time it took to train and predict using a LANDMark (No Oracle) and LANDMark (Oracle) classifier, and RF was measured.

2 $$Balanced \, Accuracy \, Score=\frac{1}{n}{\sum }_{i}^{n}\frac{{c}_{ii}}{max({{\sum}c}_{i,}, {\sum}{c}_{,i})}$$

3 $$MCC=\frac{TP\bullet TN-FP\bullet FN}{\sqrt{\left(TP+FP\right)\left(TP+FN\right)\left(TN+FP\right)\left(TN+FN\right)}}$$

### Assessment of the LANDMark, extra trees, and random forest decision space

Kappa-Error diagrams, a popular method used to analyze ensemble diversity, were constructed. These diagrams visualize the average error (y-coordinate) and agreement (x-coordinate) between pairs of classifiers in an ensemble. This information can then be used to help infer how the ensemble behaves [69]. These diagrams are created by constructing a contingency table from each pair of classifiers and calculating the average error of the pair (Eq. 4). The inter-rater agreement, measured using Cohen’s Kappa, is also calculated for each pair. These are then plotted on the Kappa-Error diagram. Finally, we examined how differences in the distribution of co-occurrences between pairs of samples in each tree differed between the four tree-based models. To do this we constructed a similarity matrix, which is simply a count of how often pairs of samples co-occur in the terminal leaves of each tree [8, 13]. These counts were then divided by the total number of trees in the forest. To create a dissimilarity matrix the square root of each frequency was taken after it was subtracted from one [8, 13, 74]. A principal coordinates analysis (PCoA) of the dissimilarities was used for visualizing and analyzing these differences. This analysis can be used to investigate the decision function of each tree-based ensemble [8, 13].4

### Analysis of metabarcoding data

A model’s ability to generalize and identify a consistent set of predictive features in amplicon sequencing data is important. We measured if perturbations of the sample space influenced generalization performance, and if these perturbations influenced the selection ASVs when using recursive feature elimination (RFE) [75]. As previously discussed, rare ASVs were removed from each raw dataset. Each dataset was then split randomly into two stratified halves. The same type of model was trained on each half and the balanced accuracy scores for each full model were calculated using the half of the data which was not used for training. RFE was used to identify the best 100 ASVs in each half of the data. A pair of reduced models using these 100 ASVs were then trained and balanced accuracy scores for each model were calculated as described above. This was repeated thirty times using different random splits. For reproducibility, the random state for each split was set to the iteration number. A within subjects repeated measures two-way ANOVA was then run to determine test for differences in the generalization performance between classifiers before and after feature selection [73]. If a statistically significant difference (*p* ≤ 0.05) was detected, a post-hoc pairwise comparison using the Bonferroni correction was conducted. Each amplicon was analyzed separately using IBM SPSS Statistics 26. Finally, for each amplicon, we created thirty different train-test splits such that the training set was comprised of 80% of the data. The generalization performance for each split was recorded and a statistical analysis carried out as described above.

The feature importance scores for each pair of reduced models were estimated using the ‘shap’ package [19]. For the linear, RF, and ETC models, Shapley scores were calculated using the ‘Explainer’ function. The algorithm parameter was set to ‘tree’ for RF and ET models. ‘KernelExplainer’ using 768 samples was used for LANDMark models. The mean absolute value of the Shapley scores was then taken and used as a proxy for each ASV’s importance. Eliminated ASVs were assigned an importance score of 0. These scores were then converted into ranks and the correlation between the two sets of ranks was calculated using Spearman’s rho [75]. Consistently higher correlations would suggest that a predictive model can consistently rank ASVs when the sample space is perturbed. A within subjects repeated measures ANOVA (followed by pairwise comparisons using the Bonferroni correction if a significant result was found) was used to determine if correlation scores between classifiers differed [73]. Each amplicon was analyzed separately using IBM SPSS Statistics 26.

To analyze how well RFE identified the same ASVs across models, we used the Jaccard distance to calculate the dissimilarity between the sets of ASVs identified using RFE. This was repeated at the genera and family levels. PerMANOVA was then run to assess if any differences could be detected [76]. We also identified the ASVs associated with the best performing fold for each model. The ‘shap’ package was used to visualize how ASVs impacted each model’s predictions. Finally, these ASVs were also compared to those identified using an indicator species analysis that was conducted in R (version 3.5) using the ‘multipatt’ function found in the ‘indicspecies’ package (version 1.7.8) [77]. The same training data used to construct each machine learning model was used in the indicator species analysis and the Benjamini–Hochberg correction was applied to correct for potential false-discoveries. To visualize overlaps between methods, Venn diagrams were created using ‘matplotlib-venn’ in Python 3.8. The F230 and BE datasets were analyzed separately.

## Results

### LANDMark performs competitively in synthetic and toy data

Friedman tests detected statistically significant differences between classifiers in each of the synthetic experiments in both the score and MCC measures (Additional file 1: Table S1). Summary statistics and location of statistical differences, using pairwise Nemenyi tests, between LANDMark (Oracle) and other classifiers are summarized in Table 2. When trained on presence-absence datasets, no classifier was able to achieve an acceptable false-discovery rate under the testing conditions. When the number of true features is very small (25 true features), we observed that LANDMark (with and without the Random Oracle enabled) consistently had higher balanced accuracy score than their peers. Between the two models LANDMark (Oracle) appeared to produce better models, although this difference was not statistically significant in any of the tests. However, some pairwise comparisons involving LANDMark (Oracle) and other models did result in statistically significant differences at the 0.05 threshold in LANDMark (Oracle)’s favor (Table 2). While other models were able to close this difference as the number of informative features increased, LANDMark (Oracle) remained as the best performing model. Generally speaking, LANDMark’s feature selection performance was on-par with other models. In only one instance was LANDMark (Oracle) statistically different than a competitor, a result which favored Logistic Regression in synthetic data constructed using 50 informative features. A full reporting of results can be found in Additional file 2.

**Table 2:** Overview of the performance of each model in each of the synthetic tests

		LANDMark (oracle)	LANDMark (no oracle)	Extra trees	LinearSVC	Logistic regression	Random forest	Ridge regression	SGD (MH)	SGD (SH)
*25 Features*	*Score*	**0.817 ± 0.183 (1)**	0.805 ± 0.167 (2)	0.405 ± 0.164 (6)*	0.672 ± 0.188 (3)	0.626 ± 0.205 (5)	0.378 ± 0.225 (8)*	0.660 ± 0.178 (4)	0.341 ± 0.146 (9)*	0.386 ± 0.170 (7)*
	*MCC*	0.678 ± 0.084 (5.5)	0.674 ± 0.083 (7)	**0.738 ± 0.7061 (1)**	0.678 ± 0.060 (5.5)	0.730 ± 0.067 (2)	0.691 ± 0.071 (3)	0.680 ± 0.077 (4)	0.520 ± 0.312 (9)	0.526 ± 0.296 (8)
50 Features	*Score*	**0.912 ± 0.127 (1)**	0.909 ± 0.112 (2)	0.713 ± 0.169 (7)	0.812 ± 0.142 (4)	0.800 ± 0.147 (5)	0.714 ± 0.172 (6)*	0.816 ± 0.150 (3)	0.565 ± 0.135 (9)*	0.569 ± 0.174 (8)*
	*MCC*	0.736 ± 0.052 (7)	0.746 ± 0.052 (4)	0.742 ± 0.054 (5)	0.773 ± 0.040 (2)	0.794 ± 0.049 (1)**	0.652 ± 0.056 (9)*	0.751 ± 0.036 (3)	0.736 ± 0.061 (6)	0.714 ± 0.072 (8)
75 Features	*Score*	**0.964 ± 0.086 (1)**	0.959 ± 0.099 (2)	0.918 ± 0.141 (5)	0.923 ± 0.124 (4)	0.874 ± 0.166 (7)	0.883 ± 0.160 (6)	0.928 ± 0.116 (3)	0.663 ± 0.167 (9)*	0.674 ± 0.143 (8)*
	*MCC*	0.786 ± 0.045 (5)	0.786 ± 0.051 (4)	0.703 ± 0.035 (7)*	0.806 ± 0.040 (2)	**0.818 ± 0.039 (1)**	0.593 ± 0.035 (9)*	0.790 ± 0.043 (3)	0.706 ± 0.068 (6)*	0.685 ± 0.081 (8)*
Average ranking (Score)		1	2	6	3.67	5.67	6.67	3.33	9	7.67
Average ranking (MCC)		5.83	5	4.33	3.17	1.33	7	3.33	7	8

The mean and sample standard deviation are reported with the best performing model being indicated in bold text. Asterisks indicate a statistically significant difference (p ≤ 0.05). A single asterisk denotes a statistically significant difference in favor of LANDMark (Oracle) while a double asterisk indicates a statistically significant difference in favor of the alternative model

**Fig. 3:**
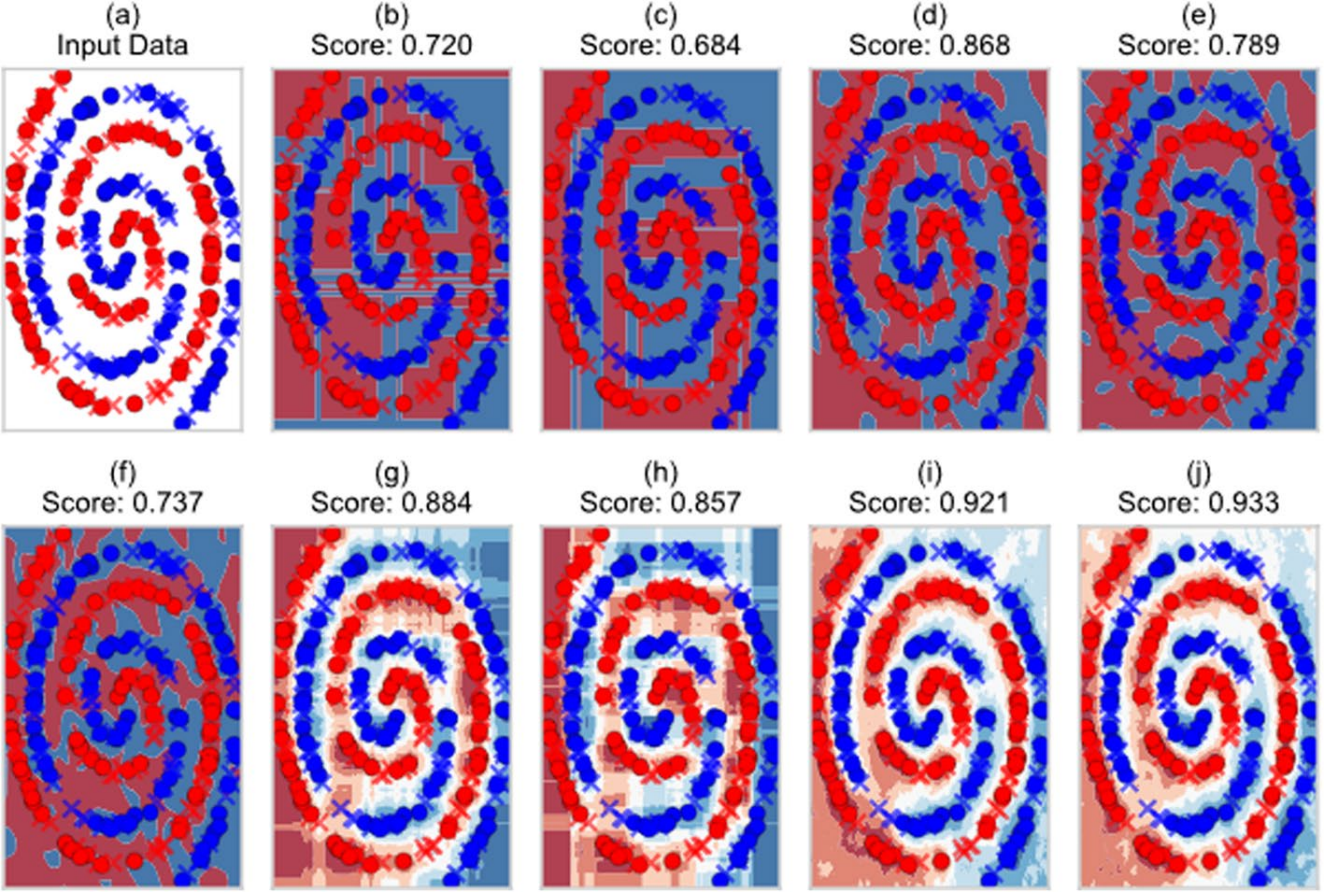
More accurate decision boundaries are recovered using LANDMark models. Decision boundaries discovered by various classifiers on two-spirals dataset. The input data (**a**) was used to train (**b**) a single Extremely Randomized Tree, **c** a single decision tree, **d**, **e** two different LANDMark (Oracle) trees that demonstrate the randomness of the algorithm, **f** a single LANDMark (No Oracle) tree, **g** an Extremely Randomized Trees classifier consisting of 100 trees, **h** a Random Forest classifier consisting of 100 trees, **i** a LANDMark (Oracle) classifier consisting of 64 trees, and **j** a LANDMark (No Oracle) classifier consisting of 64 trees. Solid circles indicate data points used for training while crosses represent validation data. The balanced accuracy of each classifier is reported as the score. The shading in each plot is a qualitative representation of how confidently each model would predict the class of a particular sample. In panels b-f the red and blue regions are not shaded and represent where each model will predict either the red or blue spiral while in panels **g**–**j** the predictions of each ensemble member are averaged. In these panels darker regions represent areas where the prediction from each model is more confident

Depending on the toy dataset, differences in generalization performance between LANDMark and its peers were observed (Table 3). However, we did not assess the statistical significance of differences within datasets since they were often related to the amount of non-linearity present in the data. For example, under these testing conditions linear classifiers will perform poorly since they will struggle to correctly identify the two classes in the ‘two spirals’ and ‘concentric circles’ datasets. Non-linear classifiers, such as LANDMark and the ET, will often do much better since they are more likely to approximate a correct decision boundary between classes (Fig. 3; Table 3). Therefore, the goal was to test if no difference in generalization performance could be detected between models across a variety of different datasets. Given this experimental setup the evidence does not support the null hypothesis. Therefore, it is likely that at least one statistically significant difference between models exists across the datasets tested in this experiment (χ^2^(8) = 41.96, *p* ≤ 1.38e−06, rho = 0.026). Nemenyi post-hoc tests revealed that most comparisons between LANDMark and other models were roughly equivalent. However, LANDMark (Oracle) did perform better than the SGD Classifier using the modified Huber and squared hinge loss functions (*p* ≤ 5.43 x 10^-4^ and *p* ≤ $3.27\times {10}^{-5}$). A full reporting of the raw data and results can be found in Additional file 3.

**Table 3:** Overview of the generalization performance (balanced accuracy) of each model when trained using toy data

	LANDMark (oracle)	LANDMark (no oracle)	Extra trees	LinearSVC	Logistic regression	Random forest	Ridge regression	SGD (MH)	SGD (SH)
Two spirals	0.935 ± 0.058 (3)	0.939 ± 0.047 (2)	**0.949 ± 0.043 (1)**	0.445 ± 0.080 (9)	0.491 ± 0.077 (6)	0.866 ± 0.062 (4)	0.495 ± 0.064 (5)	0.487 ± 0.104 (7)	0.457 ± 0.106 (8)
Concentric circles	0.844 ± 0.046 (1)	0.837 ± 0.042 (2)	0.781 ± 0.049 (3)	0.364 ± 0.116 (7)	0.428 ± 0.058 (5)	0.802 ± 0.064 (4)	0.425 ± 0.060 (6)	0.305 ± 0.079 (9)	0.348 ± 0.082 (8)
Parkinson’s disease	0.718 ± 0.082 (2)	**0.719 ± 0.093 (1)**	0.677 ± 0.076 (5)	0.679 ± 0.086 (4)	0.693 ± 0.081 (3)	0.667 ± 0.085 (6)	0.635 ± 0.080 (7)	0.554 ± 0.099 (9)	0.564 ± 0.099 (8)
Iris	0.926 ± 0.038 (3)	0.918 ± 0.037 (4)	**0.928 ± 0.037 (1)**	0.858 ± 0.076 (7)	0.890 ± 0.083 (5)	0.926 ± 0.035 (2)	0.746 ± 0.041 (9)	0.874 ± 0.068 (6)	0.796 ± 0.129 (8)
Breast cancer	**0.945 ± 0.014 (1)**	0.940 ± 0.017 (3)	0.929 ± 0.023 (7)	0.942 ± 0.014 (2)	0.938 ± 0.021 (4)	0.919 ± 0.024 (8)	0.900 ± 0.024 (9)	0.929 ± 0.021 (6)	0.931 ± 0.027 (5)
Wine	0.974 ± 0.032 (3)	0.973 ± 0.031 (5)	0.976 ± 0.032 (2)	0.967 ± 0.032 (7)	0.974 ± 0.030 (4)	0.971 ± 0.031 (6)	**0.977 ± 0.027 (1)**	0.965 ± 0.039 (8)	0.946 ± 0.046 (9)
Seeds	0.902 ± 0.022 (3)	0.905 ± 0.021 (2)	0.877 ± 0.039 (7)	0.890 ± 0.031 (4)	0.887 ± 0.022 (5)	0.849 ± 0.053 (9)	**0.931 ± 0.023 (1)**	0.874 ± 0.043 (6)	0.851 ± 0.074 (8)
Heart failure	**0.622 ± 0.057 (1)**	0.621 ± 0.064 (2)	0.557 ± 0.069 (6)	0.526 ± 0.111 (8)	0.588 ± 0.085 (4)	0.613 ± 0.061 (3)	0.574 ± 0.068 (5)	0.390 ± 0.131 (9)	0.546 ± 0.117 (7)
Cancer coimbra	**0.714 ± 0.096 (1**)	0.700 ± 0.107 (2)	0.690 ± 0.099 (3)	0.644 ± 0.124 (8)	0.580 ± 0.127 (4)	0.656 ± 0.114 (6)	0.669 ± 0.135 (5)	0.655 ± 0.121 (7)	0.623 ± 0.123 (9)
Raisin	0.839 ± 0.026 (3)	0.839 ± 0.027 (2)	0.815 ± 0.031 (8)	0.827 ± 0.049 (5)	**0.840 ± 0.034 (1)**	0.825 ± 0.032 (6)	0.832 ± 0.034 (4)	0.819 ± 0.104 (7)	0.813 ± 0.035 (9)
Two moons	0.968 ± 0.057 (2)	**0.978 ± 0.041 (1)**	0.968 ± 0.052 (3)	0.761 ± 0.169 (9)	0.803 ± 0.086 (6)	0.934 ± 0.081 (4)	0.817 ± 0.075 (5)	0.774 ± 0.090 (8)	0.778 ± 0.146 (7)
HMP	0.953 ± 0.022 (3)	0.948 ± 0.019 (7)	0.936 ± 0.025 (8)	**0.955 ± 0.021 (1)**	0.950 ± 0.018 (4)	0.923 ± 0.033 (9)	0.949 ± 0.027 (5)	0.954 ± 0.022 (2)	0.949 ± 0.026 (6)
Average Ranking	2.17	2.75	4.5	5.92 *	4.25	5.58	5.17	7 **	7.67 **

The mean, sample standard deviation, and rank for each model is reported. The best performing models are highlighted in bold text. Asterisks indicate a statistically significant (p ≤ 0.05) difference between models. A single asterisk indicates a difference in favor of LANDMark (Oracle) while a double asterisk indicates a difference in favor of either LANDMark model

The boundaries learned by individual LANDMark trees are smoother than boundaries learned by alternative models, such as those used to construct RF and ET classifiers (Fig. 3). Furthermore, it is known that individual decision trees tend to overfit, though the extent to which individual trees overfit likely depends on the dataset [8]. In the ‘concentric circles’ dataset, for example, a single LANDMark tree appears to learn the underlying data generation function while the ET and RF decision trees overfit the data (Additional file 4: Fig. S1). The opposite occurs in the spiral dataset. Only when the decisions of individual trees are aggregated do we observe that errors tend to cancel out and LANDMark models appear to learn a smoother and arguably more appropriate decision boundary (Fig. 3). Unfortunately, assessing the shapes of decision boundaries learned using higher dimensional data, which tends to be more reflective of real-world data, is more difficult.

**Fig. 4:**
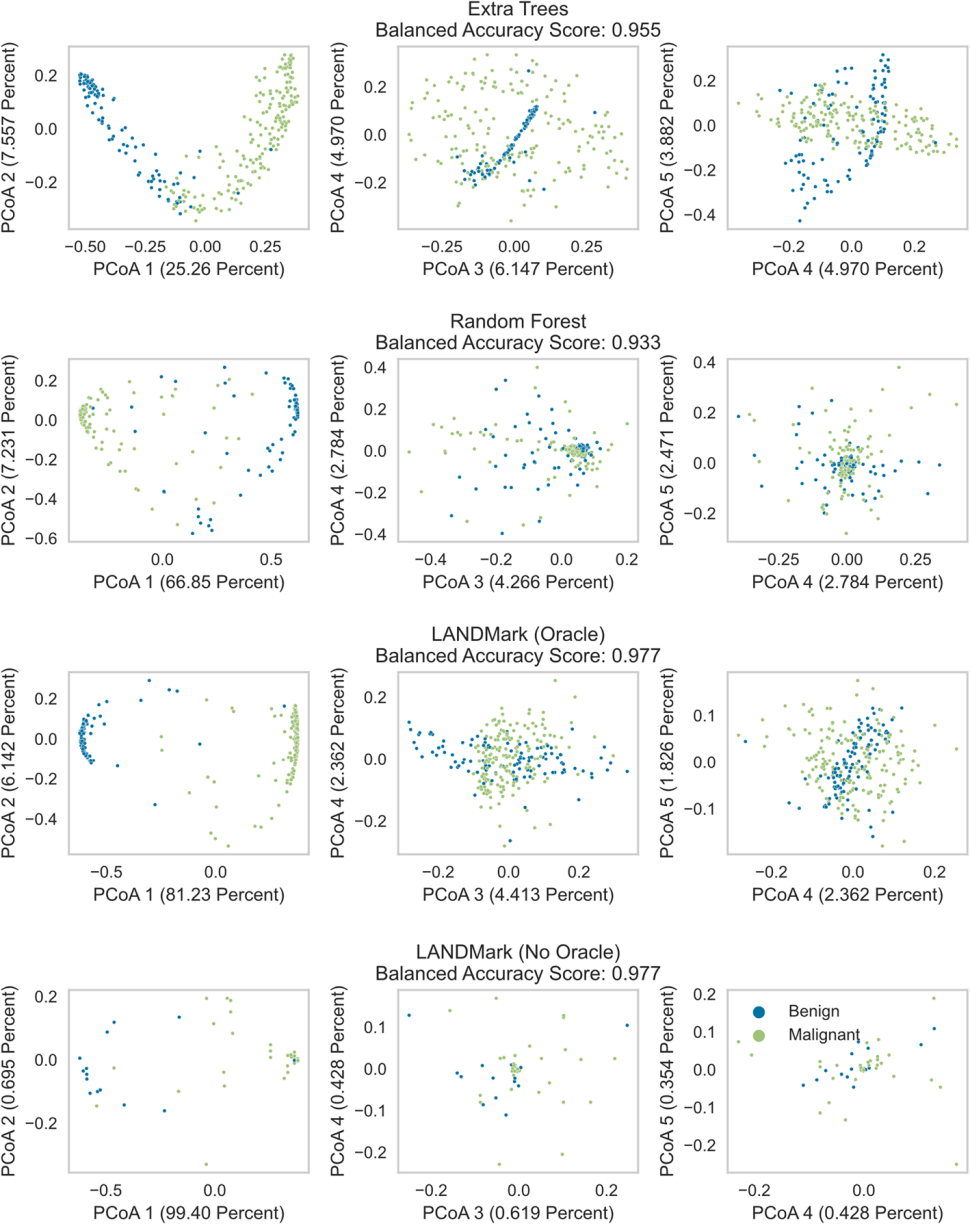
Principal Coordinate Analysis projections of test data can be used to assess model fit. Proximity matrices extracted from the Extremely Randomized Trees, Random Forest, LANDMark (Oracle), and LANDMark (No Oracle) models trained on the Wisconsin Breast Cancer dataset. Higher amounts of explained variance along the first principal component, relative to other models, reflect the ability of a model to identify a set of simple decision pathways capable of classifying samples. Higher explained variance along additional components (relative to the other models) suggest the presence of complex decision pathways and overfitting

Decision tree models, fortunately, make this task a bit simpler since the proximity matrix between samples can be extracted and visualized (Fig. 4). The results, using test samples since these reflect how well a model can generalize, are reported here. This representation shows that the first two principal axes account for a vast majority of the explained variance in each model. However, LANDMark, especially LANDMark (No Oracle), tends to capture a much greater degree of the variance within the first principal axis. Each model is also able to separate samples into two distinct regions, which is expected when using supervised learning. Together these observations suggest that each decision tree ensemble can learn a decision boundary which can effectively partition the data. The tighter groupings and greater amount of explained variance along the first component observed in the LANDMark models imply that most samples are partitioned using a similar set of rules.

### LANDMark is robust to differences in hyper-parameter settings

Our investigation into the effect of the number of estimators demonstrates that individual estimators from ET and RF classifiers perform very poorly. This is not a surprise and only confirms that individual decision trees used by these ensembles are weak learners [8, 63]. However, individual LANDMark estimators are much stronger. Further, when compared to RF and ET models, our test shows that LANDMark ensembles become more consistent classifiers as the number of decision trees increases (Additional file 4: Fig. S13). The investigation also revealed that LANDMark models were able to generalize better than RFs and ETs as the number features considered at each node increased with generalization scores plateauing at 40% of features (Additional file 4: Fig. S13). In RF and ET models, increasing this parameter did not substantially improve the performance of the ensemble. When compared to LANDMark models not using the random oracle, there is a greater amount of variation in generalization scores when the random oracle is enabled. However, variation is reduced as the number of features considered at each node or the number of estimators increases. Furthermore, enabling this hyper-parameter did not result in a statistical difference in generalization performance in other synthetic, toy, and real-world data (Tables 2, 3, 4, 5, 6). Finally, the good results observed with synthetic and toy data comes at the cost of training and prediction time. In its current state LANDMark is an algorithm that may not scale well to datasets with a larger number of samples. RF models were the fastest to train, taking roughly 0.60 ± 0.01 s to train. In contrast, a LANDMark (Oracle) model takes roughly 384.02 ± 23.21 s to train and 5.7 s to make a prediction. LANDMark (No Oracle) models were the most time consuming to train, taking roughly 543.91 ± 30.63 s. Prediction for LANDMark models was also slower than RFs, roughly taking 6 s to make a prediction.

**Table 4:** Overview of the generalization performance (balanced accuracy) of each model when trained using metabarcoding data

Amplicon	LANDMark (Oracle)	LANDMark (No Oracle)	Extra Trees	Linear SVC	Logistic Regression	Random Forest	Ridge Regression	SGD (MH)	SGD (SH)
F230	**0.957 ± 0.061 (1)**^a^	0.957 ± 0.061 (2)^a^	0.940 ± 0.061 (6)	0.944 ± 0.071 (5)	0.931 ± 0.073 (7)	0.951 ± 0.066 (3)	0.944 ± 0.069 (4)	0.922 ± 0.077 (8)	0.919 ± 0.079 (9)
BE	0.963 ± 0.049 (7.5)	0.967 ± 0.048 (5)	0.976 ± 0.043 (2)	0.970 ± 0.0054 (4)^a^	0.970 ± 0.047 (3)	**0.98 ± 0.043 (1)**	0.963 ± 0.049 (7.5)	0.963 ± 0.055 (6)	0.934 ± 0.062 (9)

The average generalization performance and standard deviation (measured using balanced accuracy) for each classification model was calculated using test data after training each classification model on data derived from either the F230 or the BE amplicons

The best performing results are in bold

^a^Truncating to three significant digits resulted in the score rounding up to the nearest thousandth

**Table 5:** Comparison of classifier generalization performance using the BE dataset before and after recursive feature elimination

	LANDMark (oracle)	LANDMark (No oracle)	Extra trees	LinearSVC	Logistic regression	Random forest	Ridge regression	SGD (MH)	SGD (SH)
LANDMark (oracle)	0.971 ± 0.002 0.963 ± 0.004	0.001 ± 0.001	− 0.005 ± 0.004	0.002 ± 0.004	− 0.013 ± 0.004	− 0.008 ± 0.004	− 0.006 ± 0.002	− 0.026 ± 0.006*	− 0.018 ± 0.006
LANDMark (no oracle)	0.003 ± 0.002	0.971 ± 0.004 0.960 ± 0.004	− 0.006 ± 0.004	− 0.003 ± 0.004	− 0.013 ± 0.004	− 0.009 ± 0.004	− 0.007 ± 0.002	− 0.027 ± 0.006*	− 0.023 ± 0.005*
Extra trees	0.009 ± 0.004	0.006 ± 0.004	0.966 ± 0.004 0.954 ± 0.005	0.003 ± 0.004	− 0.008 ± 0.004	− 0.004 ± 0.003	− 0.002 ± 0.004	− 0.022 ± 0.005*	− 0.023 ± 0.005*
LinearSVC	0.016 ± 0.003**	− 0.013 ± 0.004**	0.007 ± 0.005	0.968 ± 0.003 0.947 ± 0.004	− 0.0118 ± 0.004	0.006 ± 0.005	− 0.004 ± 0.004	− 0.024 ± 0.006*	− 0.021 ± 0.005*
Logistic regression	0.017 ± 0.005**	0.014 ± 0.004**	0.008 ± 0.005	0.001 ± 0.005	0.958 ± 0.004 0.946 ± 0.005	0.004 ± 0.004	0.006 ± 0.003	− 0.014 ± 0.006	− 0.010 ± 0.006
Random forest	0.003 ± 0.004	0.000 ± 0.003	− 0.006 ± 0.003	− 0.013 ± 0.004	− 0.015 ± 0.004	0.962 ± 0.004 0.960 ± 0.004	0.002 ± 0.004	− 0.018 ± 0.006	− 0.014 + 0.006
Ridge regression	− 0.003 ± 0.004	− 0.006 ± 0.004	− 0.012 ± 0.005	− 0.019 ± 0.004*	− 0.020 ± 0.006*	− 0.006 ± 0.005	0.964 ± 0.003 0.966 ± 0.003	− 0.020 ± 0.006	− 0.016 ± 0.006
SGD (MH)	0.030 ± 0.005**	0.027 ± 0.005	0.021 ± 0.006**	0.014 ± 0.006	0.012 ± 0.006	0.027 ± 0.006**	0.033 ± 0.006**	0.944 ± 0.005 0.933 ± 0.005	0.004 ± 0.007
SGD (SH)	0.062 ± 0.013**	0.059 ± 0.013	0.053 ± 0.014**	0.046 ± 0.014	0.044 ± 0.013	0.059 ± 0.013**	0.064 ± 0.012**	0.032 ± 0.006**	0.948 ± 0.005 0.901 ± 0.012

Models were trained using data from the BE amplicon and generalization performance was measured using the balanced accuracy score. The mean performance of each model before and after recursive feature elimination can be found along the main diagonal. The upper triangle reflects the difference of means between each comparison before recursive feature elimination while the bottom triangle reflects differences in means after recursive feature elimination. A single asterisk is used to represent a statistically significant difference (*p* ≤ 0.05) in generalization performance which favors classifiers along the rows while statistically significant differences favoring the classifiers along the columns are represented using a double asterisk. The mean and standard error are reported

**Table 6:** Comparison of classifier generalization performance using the F230 dataset before and after recursive feature elimination

	LANDMark (oracle)	LANDMark (no oracle)	Extra trees	LinearSVC	Logistic regression	Random forest	Ridge regression	SGD (MH)	SGD (SH)
LANDMark (oracle)	0.932 ± 0.005 0.933 ± 0.006	0.001 ± 0.003	− 0.008 ± 0.005	− 0.016 ± 0.007	− 0.005 ± 0.005	− 0.009 ± 0.006	− 0.008 ± 0.004	− 0.017 ± 0.007	− 0.037 ± 0.007*
LANDMark (no oracle)	− 0.001 ± 0.004	0.933 ± 0.006 0.935 ± 0.005	− 0.009 ± 0.005	− 0.016 ± 0.007	− 0.006 ± 0.004	− 0.10 ± 0.006	− 0.008 ± 0.004	− 0.018 ± 0.007	− 0.038 ± 0.007*
Extra trees	0.013 ± 0.006	0.014 ± 0.005	0.924 ± 0.005 0.921 ± 0.005	− 0.008 ± 0.007	0.002 ± 0.005	− 0.002 ± 0.005	0.000 ± 0.005	− 0.009 ± 0.007	− 0.029 ± 0.008*
LinearSVC	0.007 ± 0.006	0.008 ± 0.004	− 0.006 ± 0.007	0.916 ± 0.007 0.926 ± 0.005	0.010 ± 0.006	0.006 ± 0.009	0.008 ± 0.006	− 0.001 ± 0.009	− 0.021 ± 0.008
Logistic regression	0.012 ± 0.005	0.014 ± 0.004	0.000 ± 0.005	0.005 ± 0.006	0.927 ± 0.005 0.921 ± 0.004	− 0.004 ± 0.005	− 0.002 ± 0.004	− 0.012 ± 0.006	− 0.032 ± 0.007*
Random forest	0.011 ± 0.004	0.013 ± 0.005	− 0.001 ± 0.004	0.004 ± 0.007	− 0.001 ± 0.004	0.922 ± 0.006 0.922 ± 0.006	0.002 ± 0.005	− 0.007 ± 0.007	− 0.027 ± 0.010
Ridge regression	0.006 ± 0.004	0.008 ± 0.004	− 0.006 ± 0.005	− 0.001 ± 0.007	− 0.006 ± 0.005	0.005 ± 0.005	0.924 ± 0.005 0.927 ± 0.006	− 0.009 ± 0.006	− 0.029 ± 0.007*
SGD (MH)	0.037 ± 0.009**	0.038 ± 0.008	0.024 ± 0.010	0.030 ± 0.008**	0.025 ± 0.008	0.031 ± 0.008**	0.009 ± 0.006	0.915 ± 0.008 0.896 ± 0.008	− 0.020 ± 0.010
SGD (SH)	0.063 ± 0.012**	0.064 ± 0.011**	0.050 ± 0.010**	0.055 ± 0.012**	0.050 ± 0.011**	0.056 ± 0.010**	0.029 ± 0.007**	0.020 ± 0.010	0.895 ± 0.008 0.871 ± 0.10

Models were trained using data from the F230 amplicon and generalization performance was measured using the balanced accuracy score. The mean performance of each model before and after recursive feature elimination can be found along the main diagonal. The upper triangle reflects the difference of means between each comparison before recursive feature elimination while the bottom triangle reflects differences in means after recursive feature elimination. A single asterisk is used to represent a statistically significant difference (*p* ≤ 0.05) in generalization performance which favors classifiers along the rows while statistically significant differences favoring the classifiers along the columns are represented using a double asterisk. The mean and standard error are reported

### LANDMark can extract useful information from presence-absence metabarcoding data to create highly predictive models

When trained using 80% of the data, all models appear to generalize to unseen data well (Table 4). LANDMark (Oracle) demonstrated the strongest performance under these experimental conditions in the F230 dataset. To assess if this performance was statistically significant, a within subjects ANOVA was run to compare the generalization performance results between each classifier. This analysis suggested that differences may exist in the generalization performance between models (F(4.97, 144.16) = 2.99, *p* ≤ 0.014, partial eta squared = 0.093). However, a pairwise comparisons post-hoc followed by a Bonferroni correction did not find any significant difference between models. No significant differences in generalization performance between models could be detected when models were trained on the BE dataset (F(3.66, 106.17) = 1.91, *p* ≤ 0.120, partial eta squared = 0.062).

When testing if RFE had any impact on generalization performance when 50% of the training data was withheld, a within-subjects two-way repeated measures ANOVA found a potentially significant differences in the interaction between classifier and the use of RFE in both the BE dataset (F(2.43, 70.60) = 5.08, *p* ≤ 0.005, partial eta squared = 0.149) and the F230 dataset (F(3.84, 111.21) = 3.11, *p* ≤ 0.02, partial eta squared = 0.097). Due to presence of the significant interaction the main effects could not be interpreted. This necessitated the need to run a simple effects test to determine the location of any significant differences. Pairwise comparisons using a Bonferroni correction found multiple significant differences in generalization performance between classifiers before and after RFE in the BE (F(8, 22) = 12.62, *p* ≤ 0.001, partial eta squared = 0.821/F(8, 22) = 6.42, *p* ≤ 0.001, partial eta squared = 0.700) and F230 datasets (F(8, 22) = 4.90, *p* ≤ 0.001, partial eta squared = 0.640/F(8, 22) = 7.51, *p* ≤ 0.001, partial eta squared = 0.732) (Tables 5, 6). No significant degradation in generalization performance was detected when training a LANDMark (Oracle) model on ASVs selected using RFE from either the F230 or BE datasets.

The ability of models to learn consistent representations of the dataset is also important. A within-subjects repeated measures ANOVAs suggested that significant differences exist in the ability of classifiers to consistently rank features in both the BE (F(4.77, 138.23) = 190.08, *p* ≤ 0.001, partial eta-squared = 0.868) and F230 datasets (F(5.75, 166.71) = 217.59, *p* ≤ 0.001, partial eta-squared = 0.882). In the BE dataset, pairwise comparisons and a Bonferroni adjustment demonstrated that the feature rankings produced by LANDMark (Oracle) (rho = 0.400 ± 0.032) were more consistent than those produced by the ET, RF, Ridge Regression, and the SGD Classifier using the modified-Huber and squared-hinge loss functions (*p* ≤ 0.05 in all comparisons). However, LANDMark (Oracle)’s ability to rank features was less consistent than the LSV (rho = 0.443 ± 0.062, *p* ≤ 0.015) and Logistic Regression (rho = 0.450 ± 0.049, *p* ≤ 0.001) models. In the F230 dataset, LANDMark (Oracle) (rho = 0.466 ± 0.031) ranked features more consistently than the ET, LANDMark (No Oracle), RF, Ridge Regression, and the SGD Classifier using the modified-Huber and squared-hinge loss functions (*p* ≤ 0.05 in all comparisons). Like in the BE dataset, LANDMark (Oracle)’s ability to rank features was less consistent than the rankings from the LSV (rho = 0.507 ± 0.047, *p* ≤ 0.010) and Logistic Regression (rho = 0.512 ± 0.044, *p* ≤ 0.001) models.

**Fig. 5:**
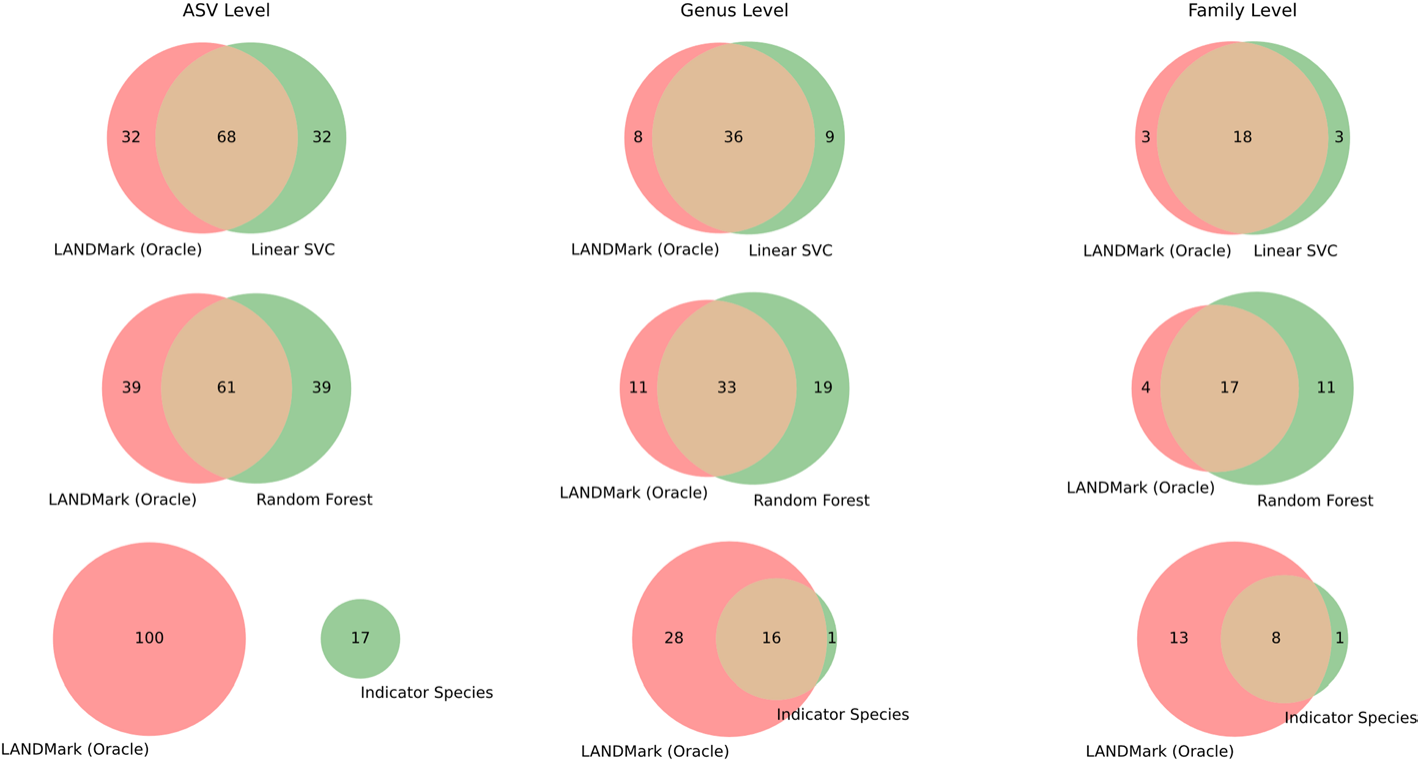
The sets of predictive ASVs selected by different classification models substantially overlap. Venn diagrams illustrating the amount of overlap in the set of ASVs (left), genera (middle), and families (right) from the Wood Buffalo F230 dataset which correspond to the fold containing the best LANDMark (Oracle) model. The top row is the comparison between the LANDMark (Oracle) and the Linear Support Vector Machine classifier (top) while the middle row compares the LANDMark (Oracle) and the Random Forest classifier. The bottom row compares LANDMark (Oracle) to an indicator species analysis

When investigating differences in how RFE selects ASVs between models, a significant PerMANOVA result was obtained at the ASV (Pseudo F = 4.38, *p* ≤ 0.001, R^2^ = 0.132/Pseudo F = 4.53, *p* ≤ 0.001, R^2^ = 0.136), genus (Pseudo F = 4.48, *p* ≤ 0.001, R^2^ = 0.134 / Pseudo F = 5.53, *p* ≤ 0.001, R^2^ = 0.161), and family (Pseudo F = 3.85, *p* ≤ 0.001, R^2^ = 0.118/Pseudo F = 5.54, *p* ≤ 0.001, R^2^ = 0.161) levels in both the F230 and BE datasets. Further investigation using pairwise comparisons between each model, followed by a Benjamini and Hochberg correction, found that most models—including LANDMark—selected unique set of ASVs, genera, and families (Additional file 1: Tables S2 and S3). Although this analysis suggests that significant differences exist between each model’s sets of ASVs each model, the effect sizes associated with these results tended to be small. When visualizing these differences, each model appears to occupy a unique region of PCoA space (Additional file 4: Figs. S2–S10). Venn diagrams demonstrate that each pair of models identifies a core set of ASVs and that there is an increasing amount of overlap between models when higher taxonomic ranks are considered (Fig. 5). A similar pattern in overlap at the genus and family levels was also observed when comparing LANDMark (Oracle) to the results of an indicator species analysis (Fig. 5). However, unlike in machine learning methods, the sets of ASVs discovered between LANDMark (Oracle) and the indicator species analysis were disjoint.

**Fig. 6:**
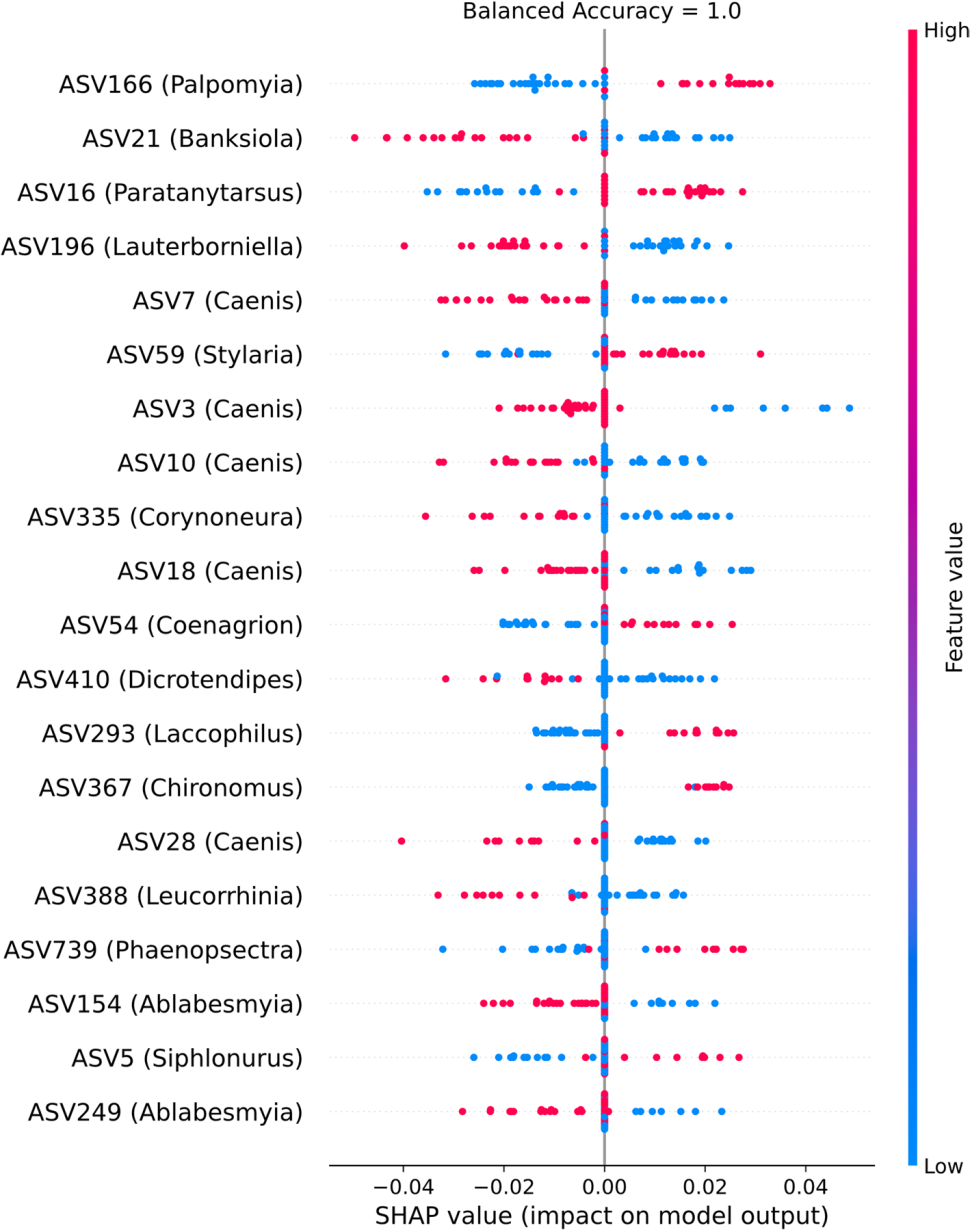
The top 20 ASVs, selected using recursive feature elimination and LANDMark, from the F230 subset. These ASVs are used by LANDMark to help identify the Athabasca or Peace River Delta. The KernelSHAP method was used to calculate the SHAP values for each ASV in each sample. Each point is a sample and the color of each point reflects the presence (pink) or absence (blue) of the ASV listed along the y-axis. The higher the absolute value of a sample’s score for a particular ASV along the x-axis, the more strongly that ASV shifts the prediction of a sample. Positive SHAP values push the prediction towards the Athabasca River Delta; negative SHAP values push prediction towards the Peace River

From the set of ASVs LANDMark (Oracle) and RFE selected, seven can be assigned to the genus *Caenis*. This suggests that the presence of this genera is important for predicting samples from the Peace River Delta (Fig. 6). Upon further investigation, these ASVs could be confidently assigned to *Caenis punctata*, a species of mayfly identified in the original study as only being found in the Peace River Delta, *Caenis youngi* and *Caenis diminuta* [47]. Our model did not identify *Caenis amica* and *Caenis latipennis*, which were identified as unique to Peace River sites in the original study, as important [47]. This difference could likely be explained by differences in methods used to assign taxonomy (MEGABLAST vs. RDP Classifier) and updates to the underlying reference database [55, 61]. Furthermore, the cutoffs used in this experiment may have been too conservative [55]. None of the identified ASVs is indicative of one river system. This contrasts with the results produced by other models, such as the LSVC and Logistic Regression (Additional file 4: Fig. S11). However, in these models ASVs are assumed to have an independent effect on their impact on the final prediction. This stands in contrast to tree-based models, where interactions between features are implicitly modelled during the construction of each tree [9, 78]. Furthermore, the Shapley scores produced using LANDMark models may reflect the effect of spatial and temporal heterogeneity on community structure [79, 80].

### LANDMark ensembles are collections of highly accurate but low diversity models

**Fig. 7:**
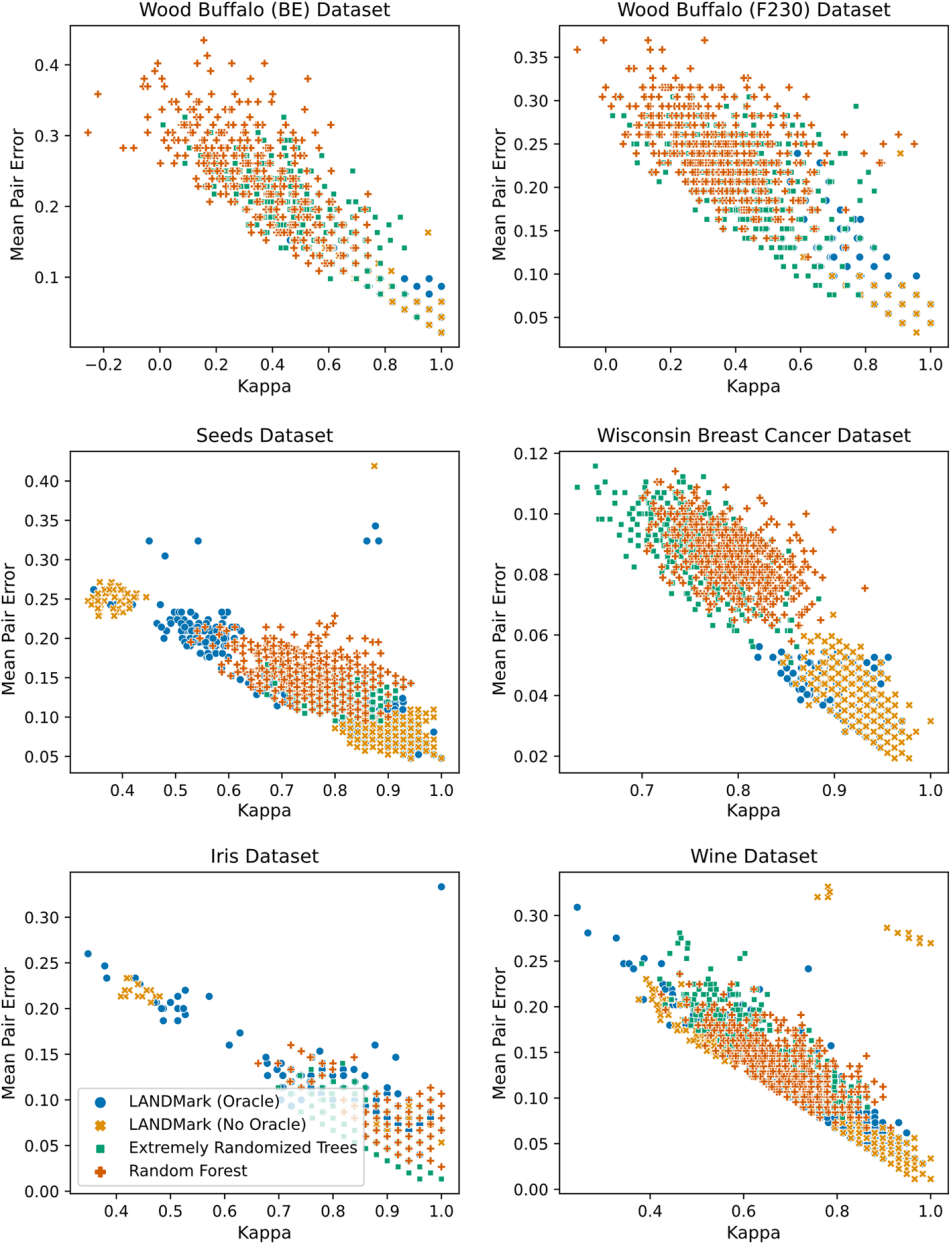
Pairwise comparisons illustrating the relationship between average error and inter-rater agreement within decision tree ensembles. Kappa-Error diagrams visualize the relationship between pairwise error (y-axis) and inter-rater reliability (x-axis) for each base estimator in LANDMark, Extremely Randomized Trees, and Random Forests. Most LANDMark trees tend to occupy the lower-right area of each plot, indicating pairs of high accuracy trees that tend to agree on classifications

Kappa-error diagrams, which visualize the relationship between mean pairwise error and inter-annotator agreement, show that LANDMark ensembles tend to produce pairs of base estimators that make fewer errors than those found in ETs or RFs (Fig. 7). However, LANDMark ensembles tend to be less diverse than their cousins. LANDMark (Oracle) models tend to be more diverse than those produced using LANDMark (No Oracle). While Kappa-error diagrams are useful in analyzing how diversity and accuracy influence classifier performance, they do not provide as much insight into how an ensemble makes decisions. For example, the decision boundaries of each base classifier in the ensemble can be very different due to randomness used to construct each node in LANDMark (Fig. 3d, e). For this reason, PCoA projections of each ensemble’s proximity matrix can be useful since these can directly visualize how decision rules impact the co-occurrence frequency [13]. In each of the tree-based models the first component accounts for the largest fraction of the variance in the proximity matrix (Fig. 4). Furthermore, we can observe that the different classes are separated into distinct regions along this component. Since these projections are made using trained models, and the balanced accuracy using the withheld data is very high for each model, these separations are a direct result of each model learning a reasonable set of rules which partition samples into their corresponding classes. Increased use of randomization in the ET and LANDMark (Oracle) reduces the amount of explained variance along the first principal component. We can also observe the presence of structures in the higher components of the ET and RF models. There also appears to be structures present in the higher components of LANDMark (Oracle). However, rotations about each axis do not reveal any discernible structure within each class.

## Discussion

We recognize that this work did not compare biomarker identification between machine learning models and generalized linear models (GLMs). While we certainly believe that this investigation is important, we felt it was necessary to devote this work to our introduction of LANDMark and discuss our motivations behind this approach to modelling amplicon sequencing data and the results of our investigation. This work is another step which further investigates the advantages and limitations of ML approaches in ecological research and is complementary to the considerable amount work done exploring GLMs. Finally, to properly frame our discussion it is important to begin with a short outline of the potential weaknesses of generalized linear models (GLMs) and other non-parametric statistical tests. Specifically, recent work has shown that violations in distributional assumptions, transformations of the data, and the models themselves can lead to inconsistent results, the misidentification of informative ASVs, and ultimately an unacceptable number of false positives and/or false negatives [7, 10, 11, 25, 29, 30, 81]. Also, while GLMs can model interactions between features and non-linearities, the extent to which this occurs is heavily dependent on the choices of the researcher and the capabilities of the software package being used [10]. Therefore, it is likely that the underlying structure of the dataset is not being adequately modelled, which places limits on the usefulness of the results [10, 11, 30].

Machine learning models, particularly ensemble models, suffer less from these limitations since most explore the underlying structure of the data and implicitly model feature interactions and non-linear relationships [7, 10, 75, 78, 82]. However, the use of machine learning models in ecological research is not as widespread when compared to other areas. For example, RF classifiers have been used in genomic research for at least 14 years and continue to be used to this day due to their ability to handle datasets containing many predictor variables and low numbers of samples [9, 63, 78, 83]. RF have been used to identify genes involved in childhood asthma, find genetic and metabolic pathways associated with phenotypes characteristic of potato quality, and this algorithm has aided the clustering of single-cell RNA-seq data [84–86]. Unfortunately, the black-box nature of most machine learning approaches and questions around ecological interpretability has hindered the application of machine learning methods to address ecological problems [31]. Although not the primary aim of our study, this work helps to address this knowledge gap by investigating the generalization and feature selection performance of some commonly used machine learning models.

It is well known that the improvements in the diversity and accuracy of base models is an effective means of improving ensemble generalization performance [87]. For example, ET introduces additional diversity by creating trees using totally random splits. This increase in pairwise diversity likely plays a role in why this algorithm tends to perform better than a RF [63]. As the pairwise-diversity between base estimators increases we should expect a decrease in the frequency of co-occurrences between similar samples. When PCoA projections of proximity matrices are created, this should manifest as a “smearing” of samples over multiple dimensions due to a reduction in explained variance along the first principal component. This is what was observed in the PCoA projections of the ET and LANDMark (Oracle) models (Fig. 4). Although there are a greater number of possible decision pathways, the additional diversity introduced by the random oracle does not appear to strongly influence the pairwise error (Fig. 7) nor does it appear to influence the overall generalization performance. These observations are consistent with previous reports which demonstrate that oblique cuts improve the accuracy of individual trees and the performance of the overall ensemble [13, 34]. Conversely, when many samples share decision pathways (co-occur more frequently) we should expect to see a tighter grouping of samples in PCoA space. This pattern was observed with RFs and in LANDMark (No Oracle) models. In RFs we believe this occurs because each node selects the best possible cut point for each feature before choosing the feature which maximizes the purity of the split [8]. A similar process is likely at work in LANDMark (No Oracle) but instead of univariate cuts, the best possible model which maximizes the purity of each split is chosen. Unlike its univariate counterpart, this process in LANDMark appears to result in the learning of smoother and simpler decision boundaries which better reflect data generating function (Figs. 3, 4).

When examining the higher components of the LANDMark PCoA projections, very little of the variation in the original proximity matrices is explained by these components and the distribution of points in this space appears to be random. This is consistent with other observations and strongly suggests that rules learned by LANDMark are more appropriate than those produced by ET and RFs [13]. The patterns found in the higher components of the LANDMark (Oracle) model appear to be directly related to the use of the random linear oracle since when this feature is disabled, as in LANDMark (No Oracle), these patterns disappear. By randomly partitioning the feature space each branch leading away from the root node of the LANDMark (Oracle) tree can be considered an independent model [35, 88]. Each branch is only capable of learning rules which partition samples above or below the hyperplane defined by the random oracle. Due to this it becomes more likely that some features will become mutually exclusive on either side of the hyperplane with the probability of an exclusion increasing as the distance from the hyperplane increases. This provides an explanation for the non-descript clouds of samples spread about each principal axis in the higher components of the LANDMark (Oracle) PCoA space. Contrary to previous work [13], we argue that this structure is not necessarily a problem because no additional structures were observed within the distribution of samples in each class. Also, if this conjecture is true, it likely explains why LANDMark (Oracle) was able to select more consistent sets of ASVs when compared to LANDMark (No Oracle). Random splits of the feature space decompose the original classification problem into two smaller subproblems [35, 88]. Supervised models trained on each subproblem are then more likely to find themselves in positions where they can learn more about the underlying structure of the data [34]. Therefore, by randomly partitioning the feature space, each independent branch in a LANDMark (Oracle) tree is more likely to identify a useful set of informative features. This interpretation is consistent with other work which has demonstrated trees constructed using learned nodes are better able to adapt to the training data and generate appropriate decision surfaces [13, 34, 35, 88].

LANDMark’s ability to search for and then selecting the best possible split function creates a diverse collection of trees and we believe that this directly translates into the performance gain over ET and RFs. Although the observed pairwise diversity of each tree decreases, we believe that there is an actual increase in the diversity because Cohen’s Kappa only captures the level of agreement between the output of two annotators and not the diversity in the underlying decisions which lead to the output of each annotator [89]. This increase in the intrinsic diversity between each individual estimator results in improvements in the independence of each tree’s output. This, along with the improvements in base estimator accuracy, reduces the overall error rate of LANDMark ensembles since incorrect classifications from one tree are more than likely to be corrected by correct classifications in others. Taken together, this analysis can provide greater insight LANDMark's inner workings and greater confidence in our model’s predictions. This is particularly important when working with medical and ecological data because any health or policy related decisions that stem from predictive models or the features identified by these models must accurately reflect the biological reality of the system. However, this analysis is not complete. To fully understand the nature of any differences between different types of samples additional work is needed. For example, a phylogenetic transformation of the data followed by a LANDMark analysis could be useful in understanding how the covariation of macroinvertebrate clades changes between environments [90]. Modifying the LANDMark algorithm to support a regression analysis could also be used to assess the chemistry of each site [82].

This work has implications when choosing, evaluating, and drawing conclusions using supervised models trained on amplicon sequencing datasets and other similar sequence data. While highly predictive models can be used to understand complex natural processes [7] it is also important to consider how various models identify important features, how consistently models identify and order features, and how the topology of the decision space reflects the selection of features. LANDMark is likely to be less influenced by distributional differences due to it being a non-parametric model of the data [8, 9, 82]. The evidence supporting this comes from the sample perturbation trials using the amplicon sequencing datasets. When trained using different perturbations of real amplicon sequencing data LANDMark models appear to be very self-consistent, although not as self-consistent as LSV and Logistic Regression models. While this may be a concern if one is looking for an absolute ordering of importance, our work demonstrates that the overall interpretation of the top performing models is unlikely to change. The ASVs identified by using RFE tend to map to a similar set of genera and families, and the effect sizes associated with statistically significant differences between sets tends to be small (Fig. 5; Additional file 1: Tables S2 and S3). Furthermore, given the highly complex nature of ecological communities, tree-based ensemble methods are more likely to accurately capture the underlying patterns which differentiate communities [9, 10]. Given this and that LANDMark often generalizes well to unseen data, a slightly lower consistency score is unlikely to be a concern. However, this does present itself as an opportunity to improve the calculation of feature importance scores in LANDMark. Finally, while model introspection has come a long way, we still cannot “peer into the black box” fully. Biases inherent to each model could also cloud potentially important relationships. Finally, we note that performance measures derived from the use of datasets with small sample sizes, such those used here, could result in an optimistic assessment of generalization performance [91]. Therefore, we should proceed with an abundance of caution when drawing broad conclusions from analyses employing machine learning models. We stress that extra care must be taken to ensure the quality of any conclusions since they can be used to help inform public policy and understand the biological factors impacting human health and disease.

In synthetic tests LANDMark’s MCC scores were competitive with other classification models, but it was never able to consistently identify the largest fraction of truly informative features. When generalization performance was measured, however, LANDMark was always the best performing model. This suggests that LANDMark may sacrifice some of its ability to detect truly informative features in exchange for learning a more predictive model. This is a particular useful characteristic since amplicon sequencing data is very sparse, can contain noise, and the number of samples is often very low [5, 11, 14].The excellent generalization performance we observed in synthetic data was mirrored in tests using the toy datasets. In these tests LANDMark models performed well and earned the best overall ranks across the datasets tested. While the performance of LANDMark in these tests is very encouraging, we do need to point out that these tests are in no way exhaustive and their likely exists data for which LANDMark is utterly unsuited [92]. We stress that when modelling a natural process using machine learning great care should be taken in the selection and evaluation of machine learning algorithms. Also, while good performance in synthetic and toy data is encouraging, the same good performance is not always guaranteed in real-world testing conditions. Our synthetic tests also show LANDMark is relatively robust to changes in its hyper-parameters. Unfortunately, while it can create very accurate models, the time it takes to model a large dataset can be a particular concern. This is a problem that does not have any easy solutions since using learned models at each node is a core feature of LANDMark. Therefore, it is necessary to investigate approaches which can minimize the impact of computationally expensive steps. Recent work with single-cell RNAseq data has shown that clustering algorithms, such as HDBSCAN, can identify internally consistent sets of clusters [93]. Summarizing large datasets using clustering is also used by the ‘shap’ package [19]. Adding a clustering step before growing each tree could be one possible approach to improving the performance of LANDMark with large datasets. Clustering can be done before each tree is grown and would be used to find smaller set of samples which approximate the global structure of the data. Finally, taking advantage of a more powerful GPU, switching to GPU accelerated algorithms, and adding hyper-parameters which allow users to control which algorithms are used could result in additional performance improvements.

Comparisons between our model, an indicator species analysis, and other machine learning models are encouraging since all methods detect a core set of genera and families. The comparisons between machine learning models are particularly interesting since there is a considerable amount of overlap between methods. This could be useful since integrating the results from multiple approaches has been suggested as a method which can find a robust subset of biomarkers capable of effectively predicting outcomes of interest [94]. Several of the identified ASVs are unique to each method and these may also carry important discriminatory information. However, without further work we speculate that these differences are likely a result of algorithmic biases. We believe this to be the case because the overlap at the genus and family level for all machine learning models was substantial and because ASVs selected by LANDMark models appear to be found in a region bounded by various linear models (Additional file 4: Fig. S2). The latter observation was not unexpected since each node in a LANDMark tree likely uses one of these models. This may allow LANDMark models to average the algorithmic biases from these models. A deeper investigation is necessary to understand this behavior, however.

Our work provides some evidence that an indicator species analysis may not be a productive approach to biomarker selection. Due to the large number of multiple comparisons in ASV datasets, this analysis is likely to be overly conservative. For example, no overlap between the best LANDMark model and an indicator species analysis trained on the same data was observed at the ASV level (Fig. 5). Although an indicator species analysis overlapped substantially with LANDMark (Oracle), only a fraction of the genera and taxa detected by both methods were shared. Furthermore, when building predictive models, we believe that an indicator species analysis cannot quantify complex interactions between ASVs and therefore cannot fully measure the predictive capacity of the ASVs which it discovers [77, 82]. For example, decision trees can capture interactions between two ASVs when classification is conditional upon one ASV appearing before the other [9, 78, 95]. Since LANDMark is a tree-based algorithm, it can also implicitly model ASV interactions in this manner. While we did not explore how this can occur, we speculate that LANDMark may be able to discover interactions between sets of ASVs (as opposed to single ASVs) since LANDMark learns splitting functions using the information from multiple ASVs. In addition, if LANDMark’s neural network model is chosen as a splitting function, ASV interactions can potentially be captured within nodes and between nodes. Finally, an indicator species analysis has been shown to be sensitive to uneven sampling of classes, the presence of missing data, and the distribution of species across classes [82]. Together, this strengthens the argument for the use of machine learning approaches when searching for bioindicators.

## Conclusions

This work has introduced LANDMark as a potential alternative to RFs for the analysis of amplicon sequencing studies. We show that LANDMark has several characteristics which make it amenable to amplicon sequencing work. Specifically, LANDMark can create highly predictive and consistent models, which is important since amplicon sequencing datasets tend to be very sparse and often contain few samples. We demonstrate that LANDMark has a distinct advantage over machine learning models, such as the Linear SVC, because it can learn decision boundaries that more accurately reflect any underlying non-linearities in the data. More generally, we also provide ecologists with an approach to analyzing the inner workings of trained machine learning models, particularly tree-based ensembles and other “black box” classifiers. By performing a careful analysis of trained machine learning models, a better understanding of how models arrive at their decisions and the potential biases associated with those decisions can be reached. Finally, while we show that trees using learned oblique and non-linear cuts can be effective tools for analyzing amplicon sequencing data, this work also highlights the weaknesses of this approach. Specifically, the amount of time it takes for the algorithm to execute can be quite high. Therefore, to exploit the advantages of complex models, such as LANDMark, it is important to further develop and fine tune our algorithm so it can handle a larger number of samples. By investigating, comparing, and understanding these methods we can develop clearer insights about the complex processes which drive the natural world.

## Supplementary Information

**Additional file 1.** Tables 1 to 4.

**Additional file 2.** Results and statistical analysis of synthetic tests.

**Additional file 3.** Results and statistical analysis of toy data sets.

**Additional file 4.** Figures 1 to 12.

**Additional file 5.** Algorithm One.

**Additional file 6.** Processed sample by ASV matrices, associated metadata, indicator species results, and statistical analyses, parameter testing results, training time results.

## Data Availability

Authors can confirm that all relevant data are included in the article and/or its supplementary information files.
