# Supplementary material for: LANDMark: an ensemble approach to the supervised selection of biomarkers in high-throughput sequencing data
Source: BMC Bioinformatics. 2022 Mar 31;23:110. doi: 10.1186/s12859-022-04631-z (PMC8969335; doi:10.1186/s12859-022-04631-z)

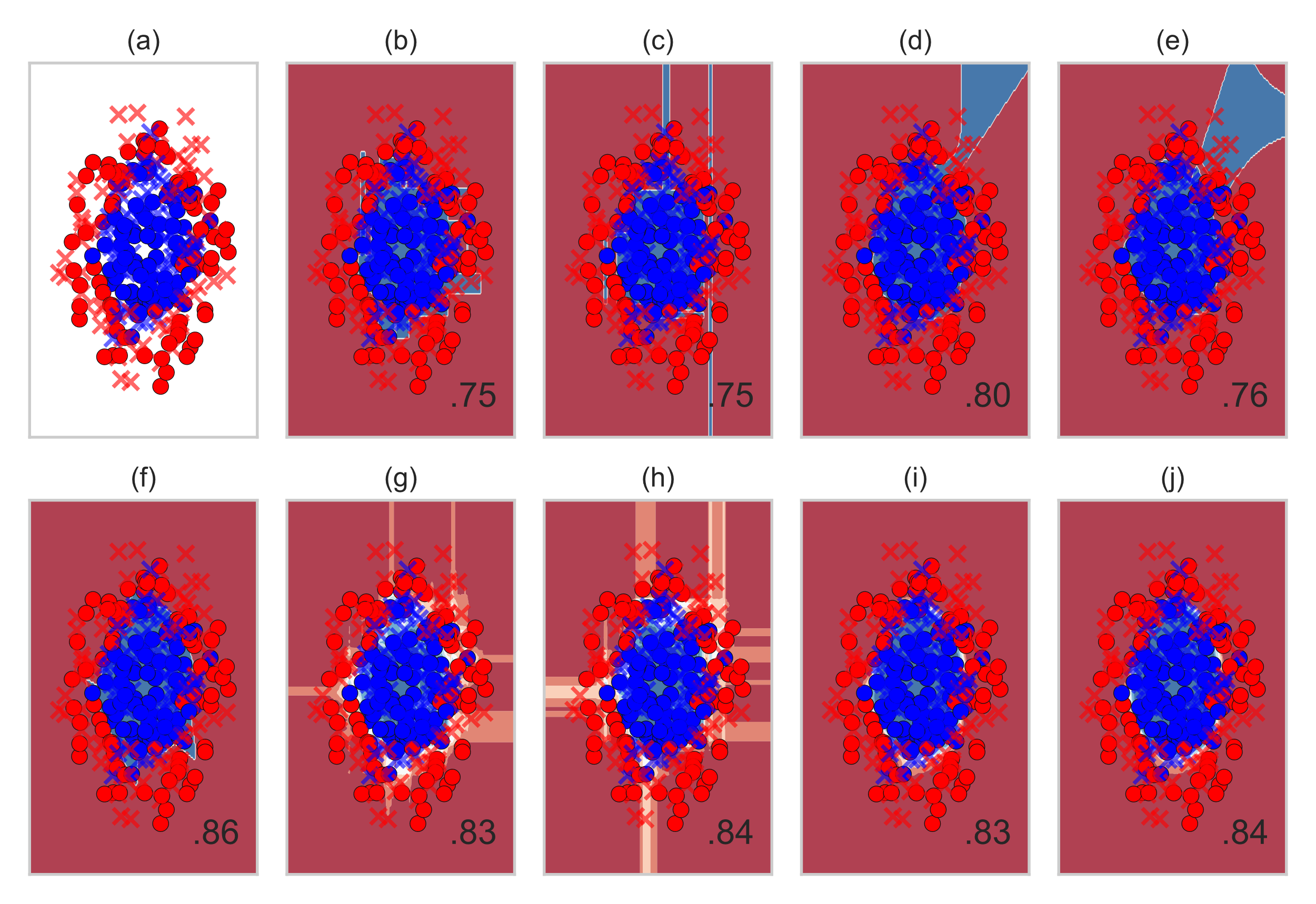


Suppl Figure 1: Decision boundaries discovered by various classifiers on concentric circles dataset. The input data (a) was used to train (b) a single Extremely Randomized Tree, (c) a single decision tree, (d and e) two different LANDMark (Oracle) trees, (f) a single LANDMark (No Oracle) tree, (g) a full Extremely Randomized Trees classifier consisting of 100 trees, (h) A full Random Forest classifier consisting of 100 trees, (i) a LANDMark (Oracle) classifier consisting of 64 trees, and (j) a full LANDMark (No Oracle) classifier consisting of 64 trees. Solid circles indicate data points used for training while crosses represent validation data. The accuracy of each classifier is reported in the bottom right. In each graph, darker areas represent regions of greater confidence.


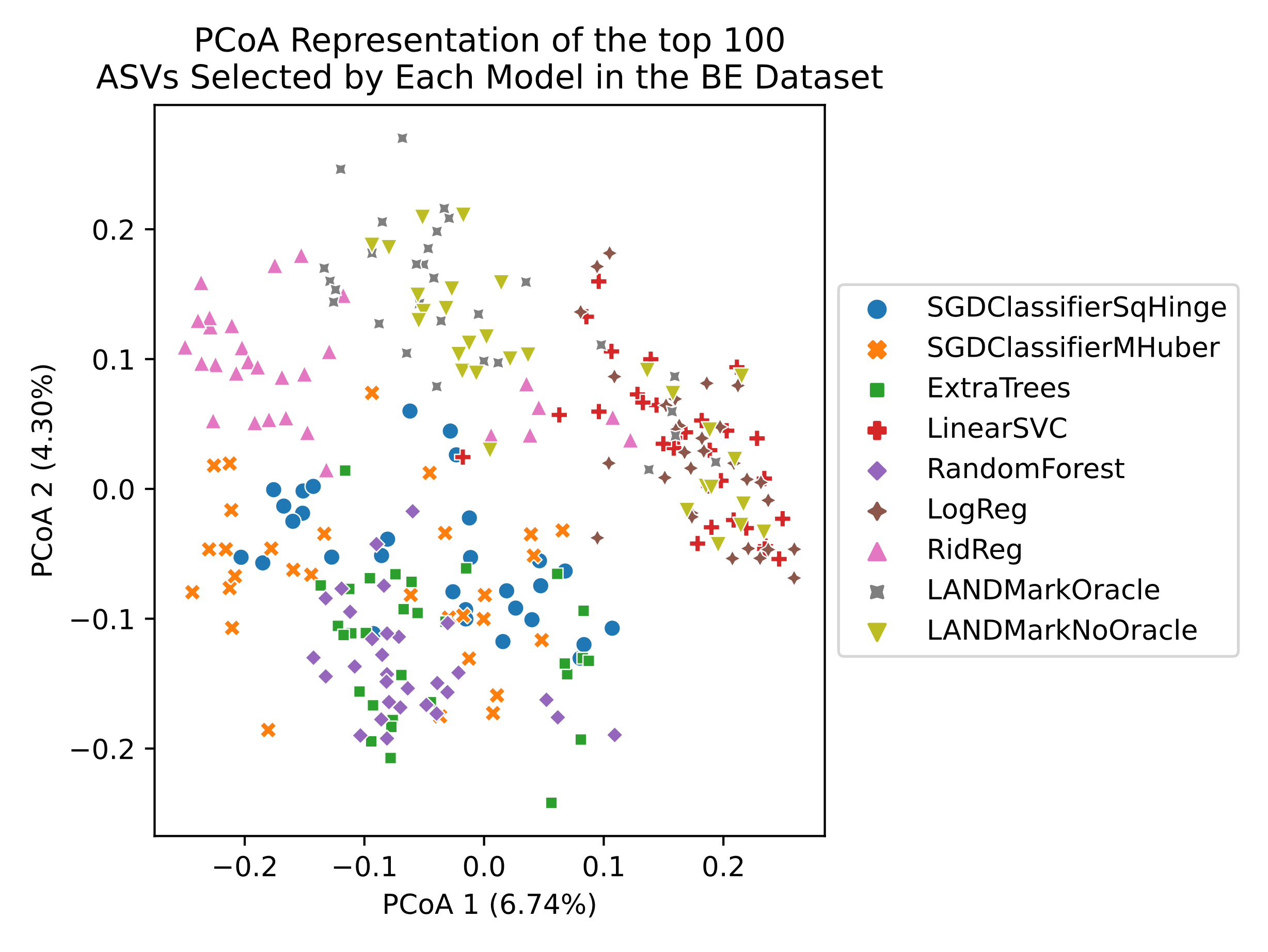


Suppl Figure 2: PCoA projection of each of the top 100 ASVs selected by each model after recursive feature elimination. Models were trained on the dataset derived from the BE amplicon. Linear models appear to occupy unique regions of PCoA space. LANDMark models appear to occupy a region surrounded by the various linear models.


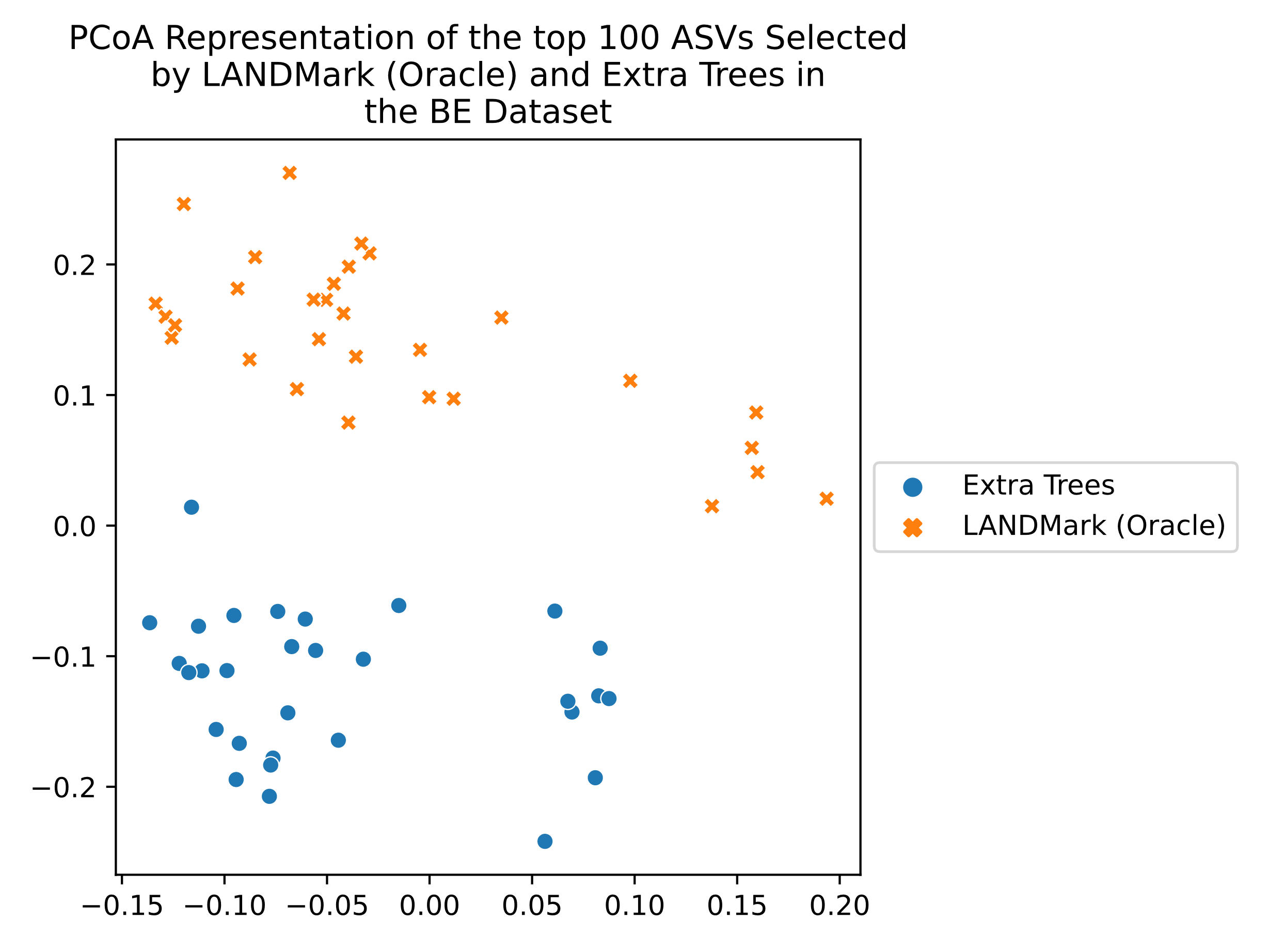


Suppl Figure 3: PCoA projection of each of the top 100 ASVs selected by LANDMark (Oracle) and the Extra Trees classifiers after recursive feature elimination. Models were trained on the dataset derived from the BE amplicon.


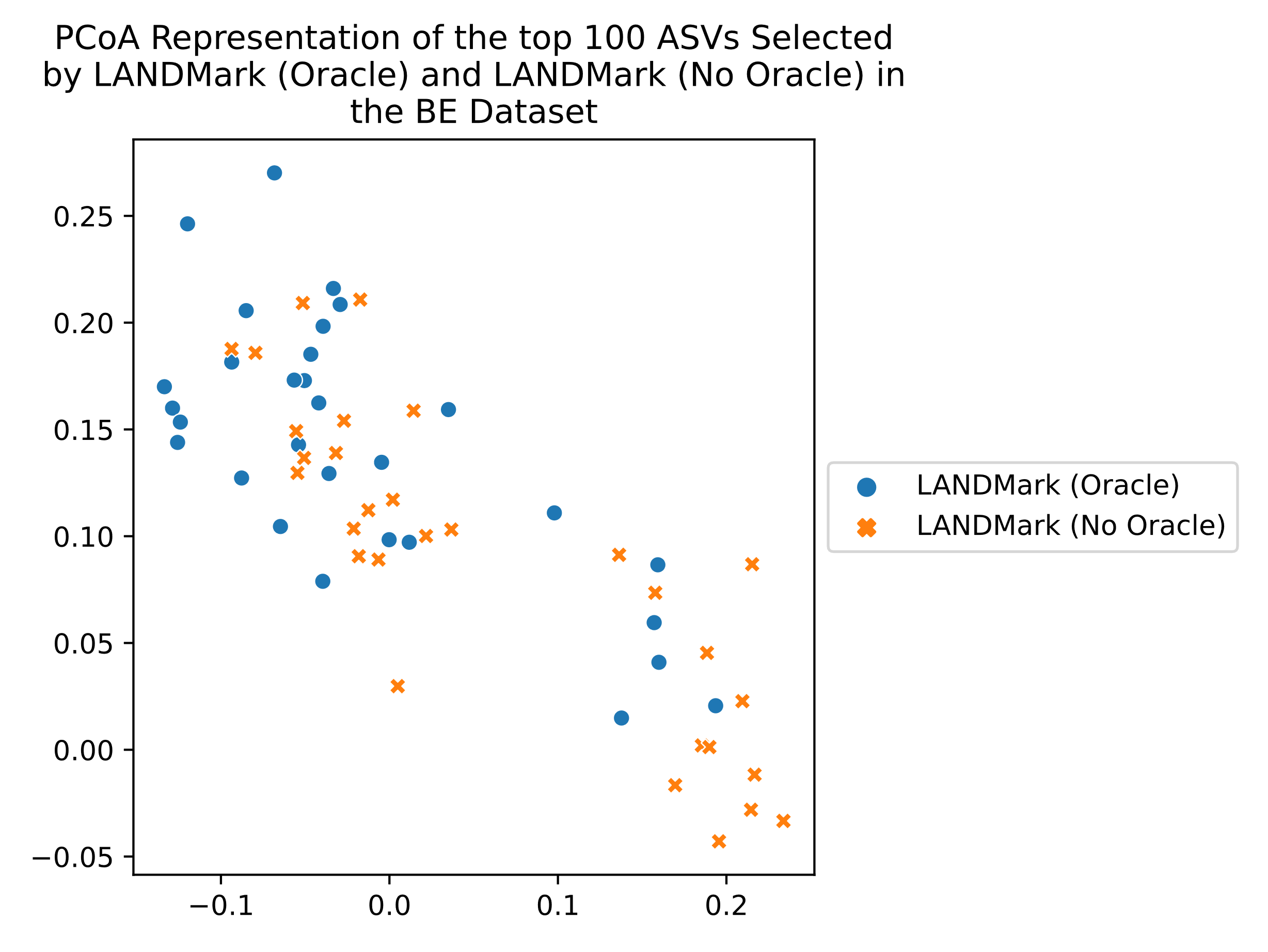


Suppl Figure 4: PCoA projection of each of the top 100 ASVs selected by LANDMark (Oracle) and the LANDMark (No Oracle) classifiers after recursive feature elimination. Models were trained on the dataset derived from the BE amplicon.


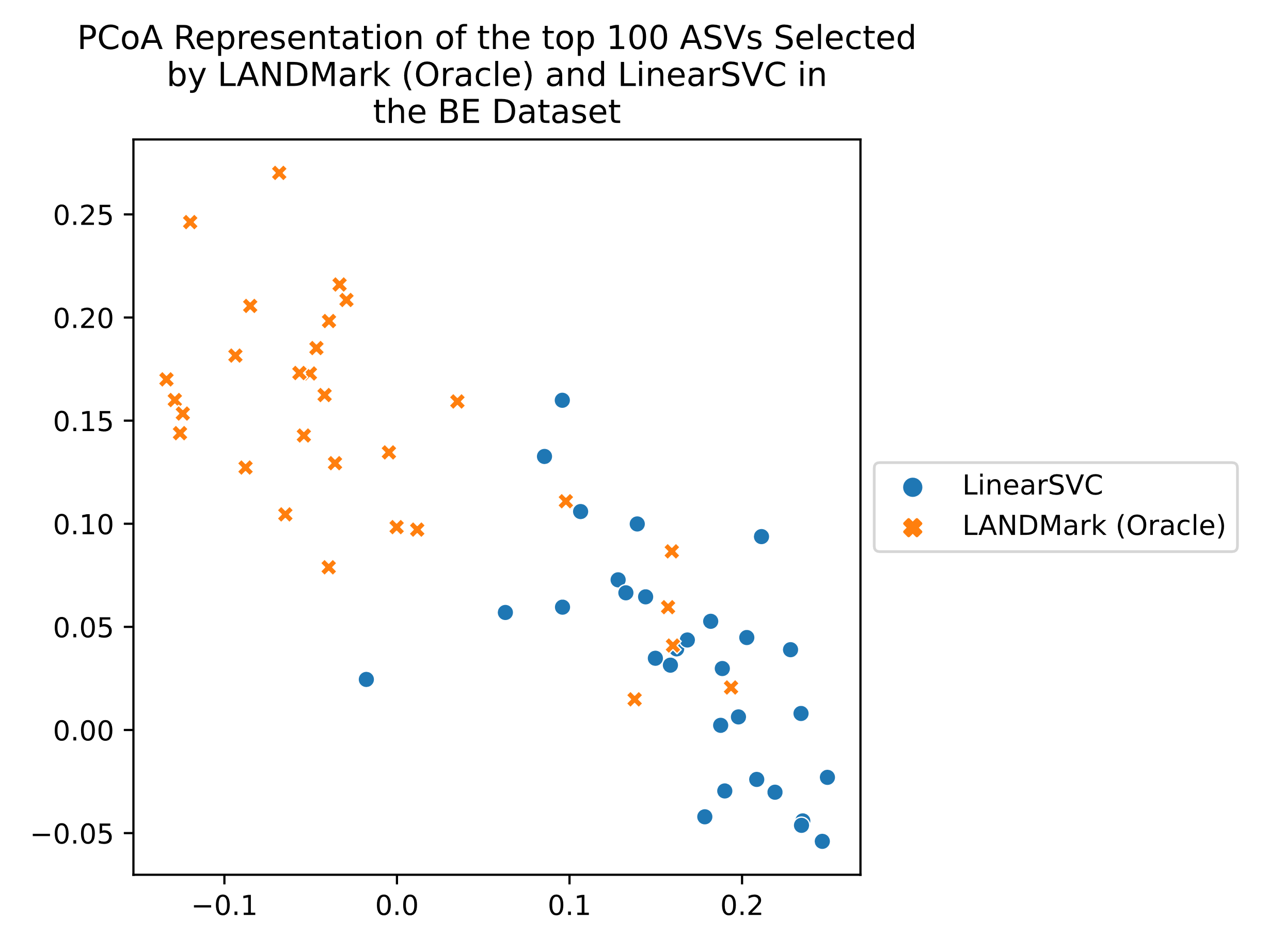


Suppl Figure 5: PCoA projection of each of the top 100 ASVs selected by LANDMark (Oracle) and the Linear SVC classifiers after recursive feature elimination. Models were trained on the dataset derived from the BE amplicon.


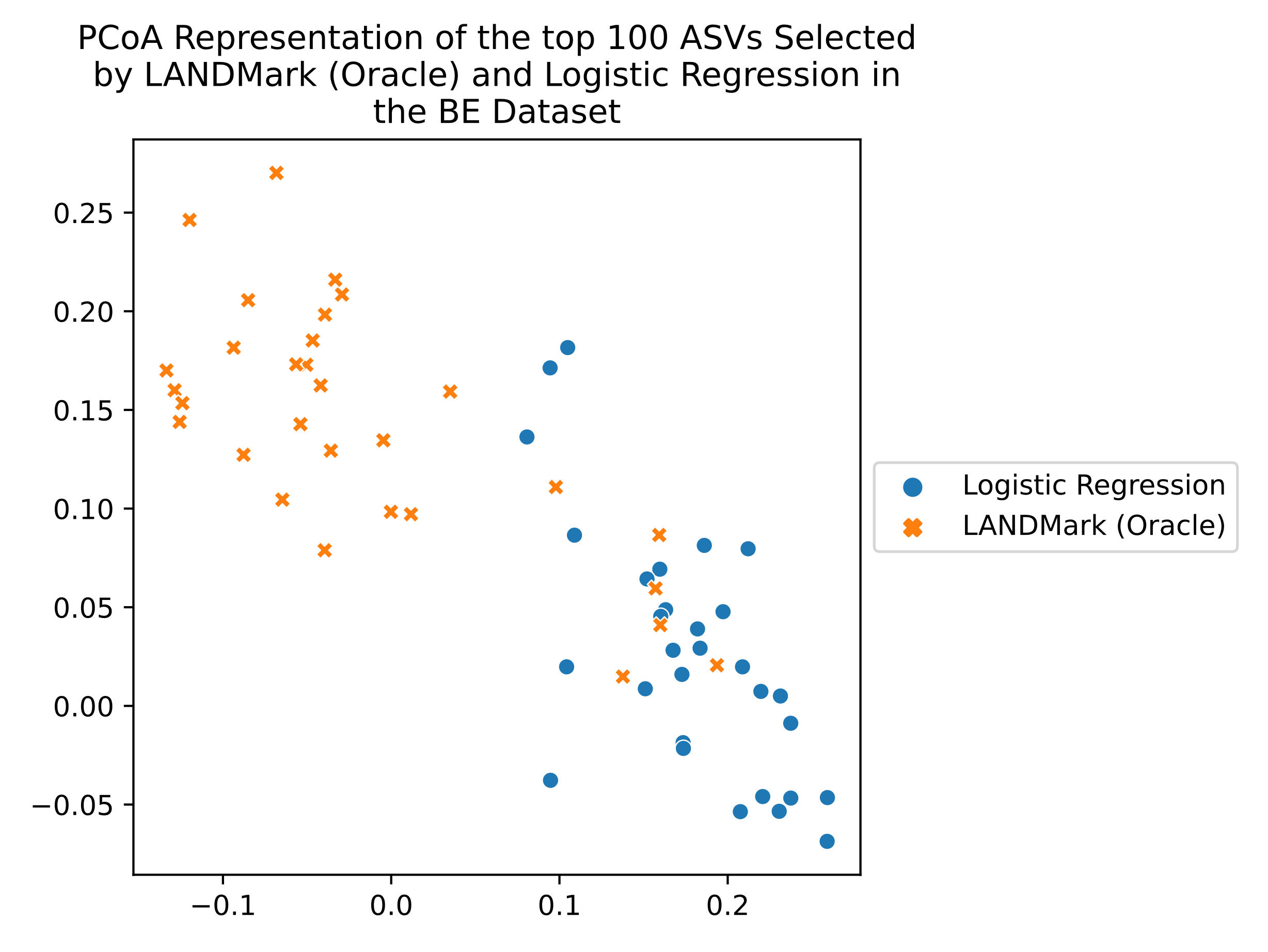


Suppl Figure 6: PCoA projection of each of the top 100 ASVs selected by LANDMark (Oracle) and the Logistic Regression classifiers after recursive feature elimination. Models were trained on the dataset derived from the BE amplicon.


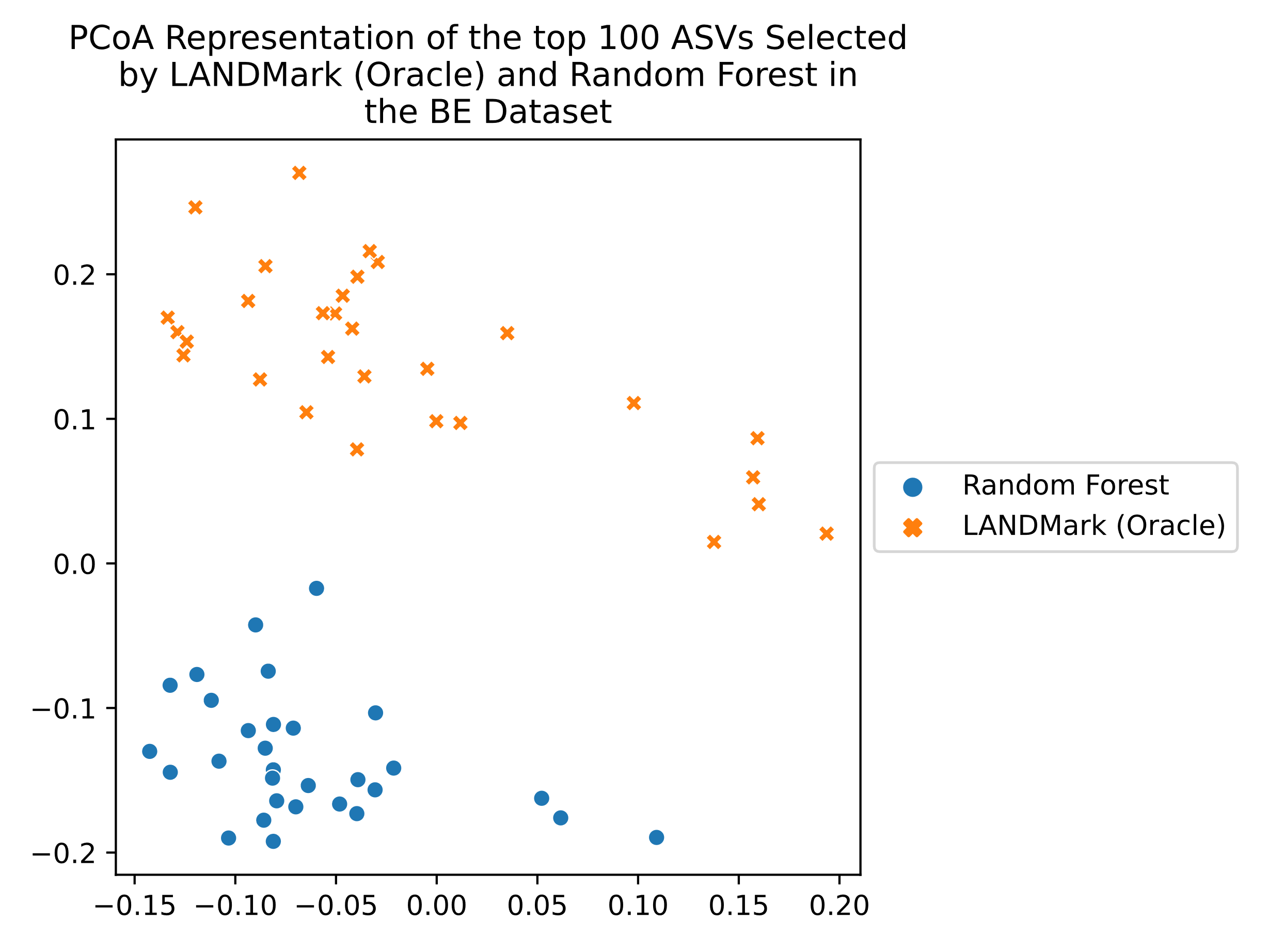


Suppl Figure 7: PCoA projection of each of the top 100 ASVs selected by LANDMark (Oracle) and the Random Forest classifiers after recursive feature elimination. Models were trained on the dataset derived from the BE amplicon.


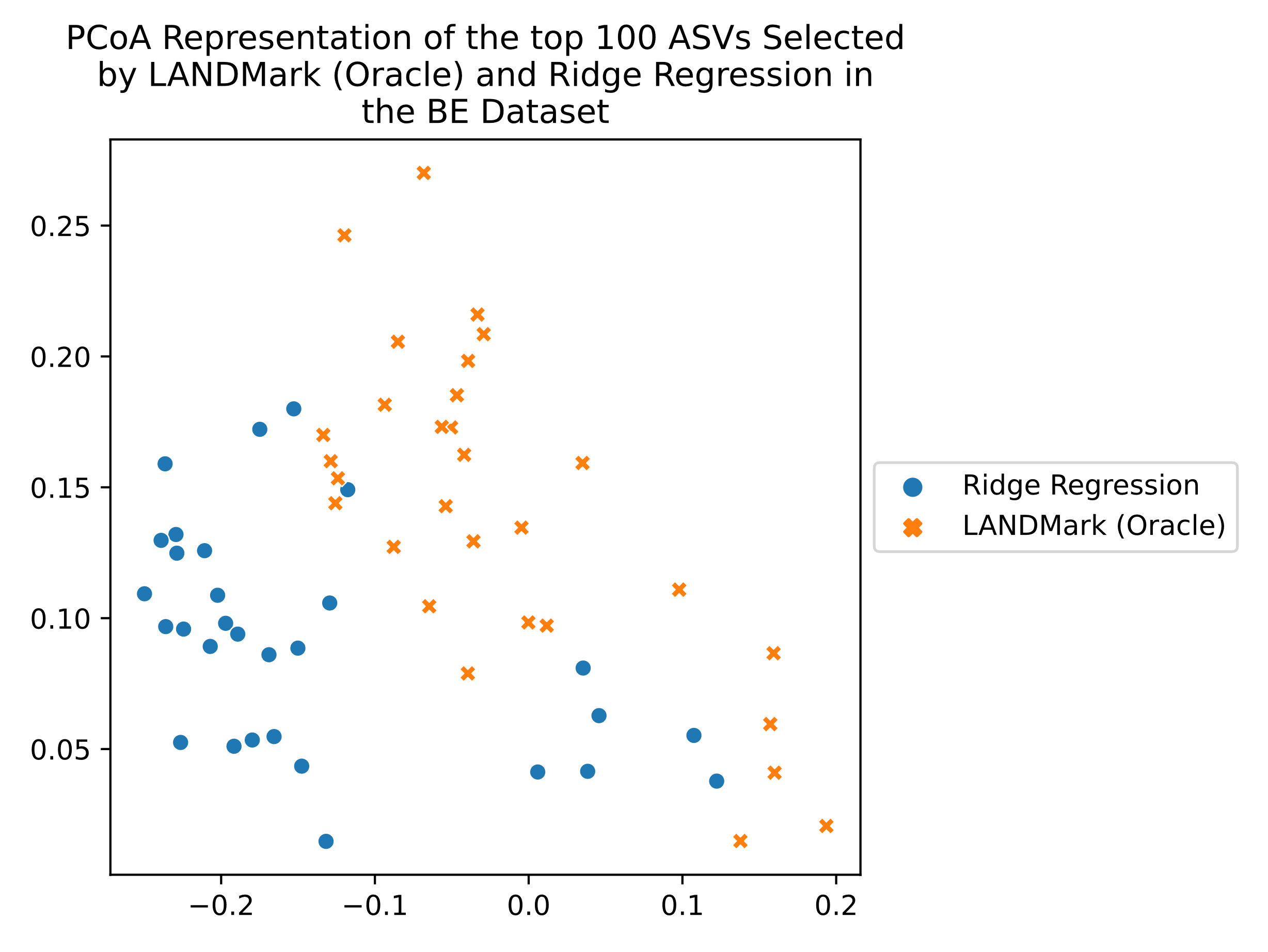


Suppl Figure 8: PCoA projection of each of the top 100 ASVs selected by LANDMark (Oracle) and the Ridge Regression classifiers after recursive feature elimination. Models were trained on the dataset derived from the BE amplicon.


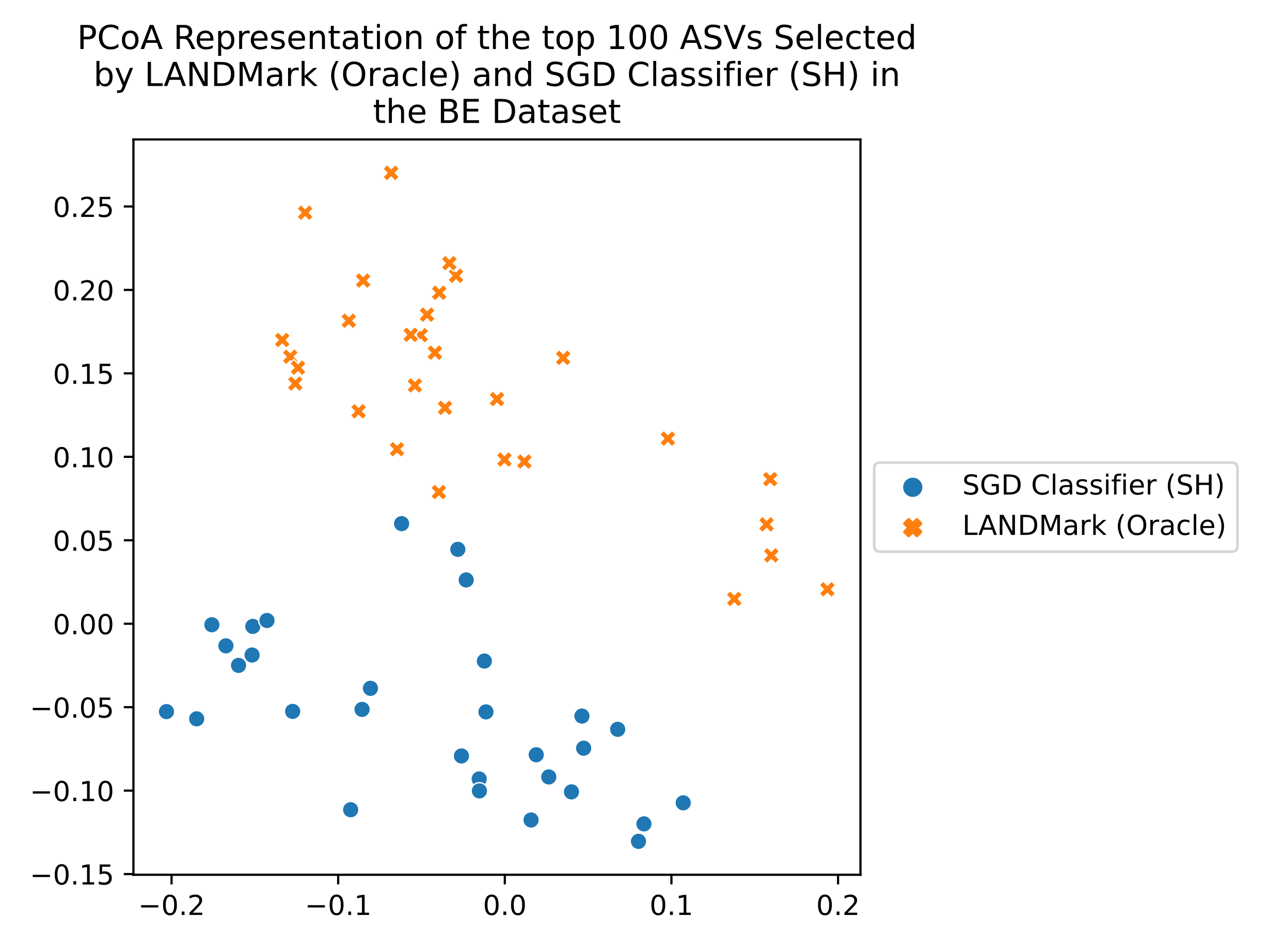


Suppl Figure 9: PCoA projection of each of the top 100 ASVs selected by LANDMark (Oracle) classifier and the SGD classifier using the squared-hinge loss function after recursive feature elimination. Models were trained on the dataset derived from the BE amplicon.


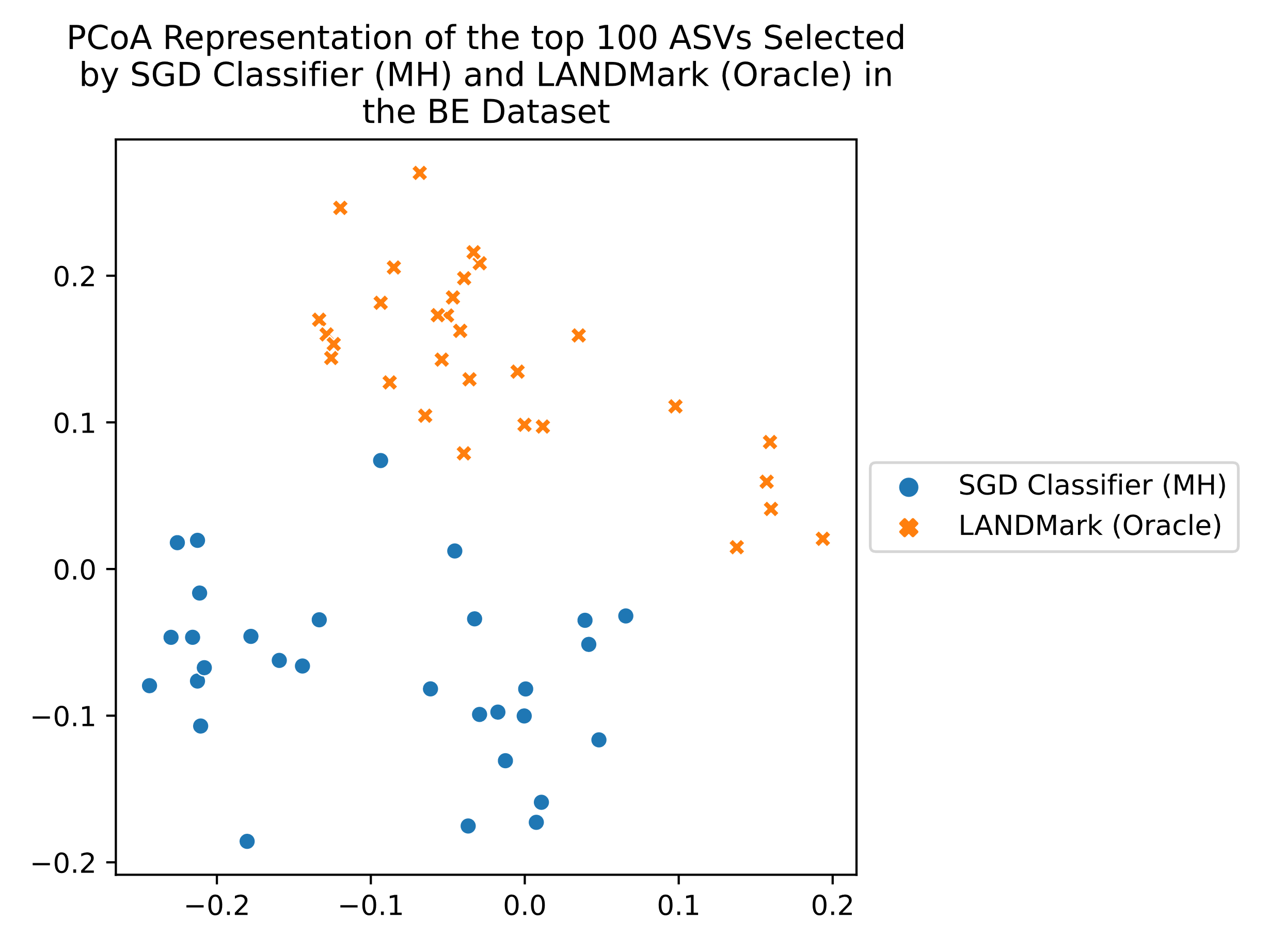


Suppl Figure 10: PCoA projection of each of the top 100 ASVs selected by LANDMark (Oracle) classifier and the SGD classifier using the modified-Huber loss function after recursive feature elimination. Models were trained on the dataset derived from the BE amplicon.


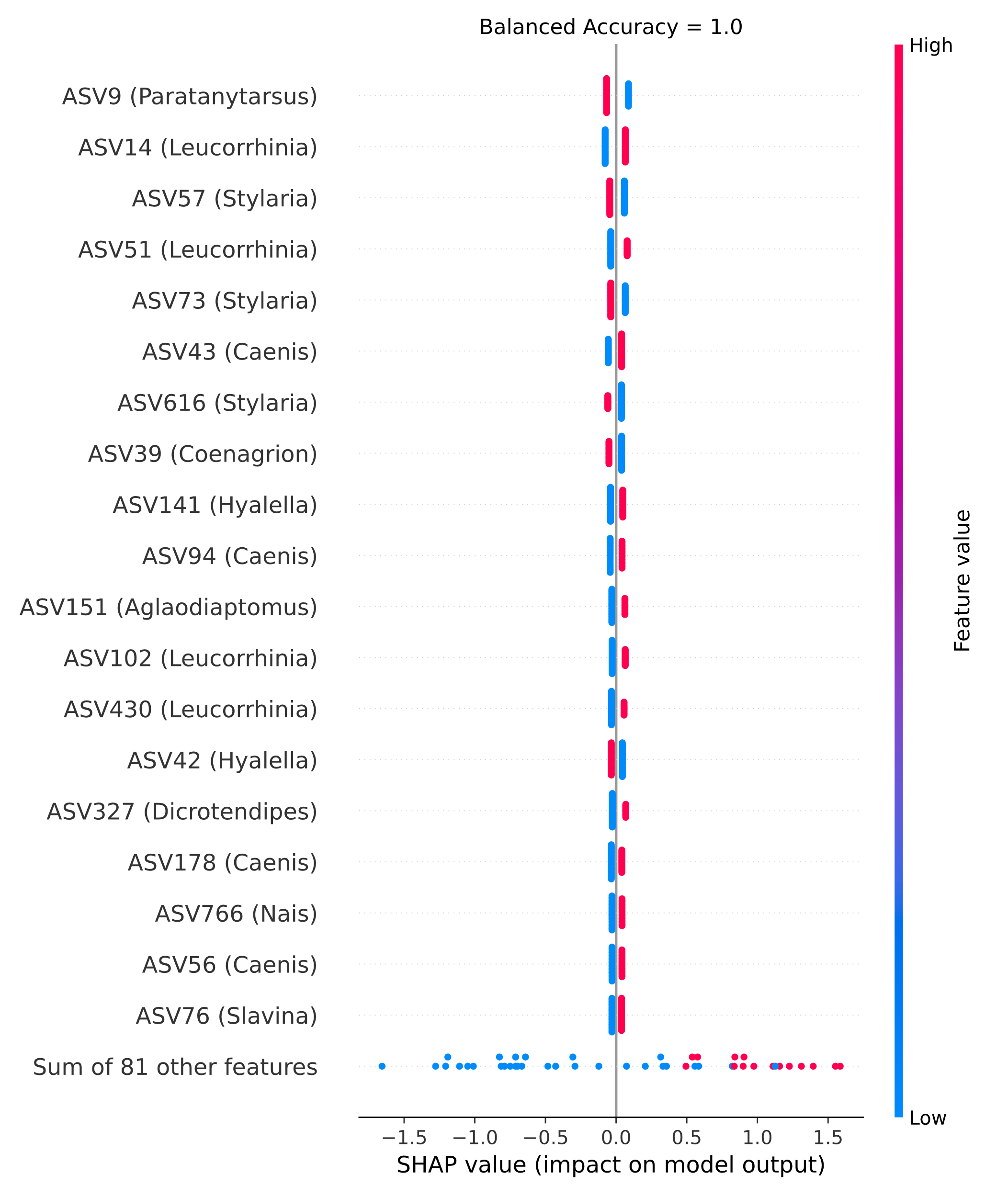


Suppl Figure 11: A list of ASVs, selected by using recursive feature elimination and Logistic Regression, which are used by Logistic Regression to help identify the Athabasca or Peace River Delta. The KernelSHAP method was used to calculate the SHAP values for each ASV in each sample. Each point represents on this graph is a sample., The color of each point reflects the presence (pink) or absence (blue) of the ASVs along the y-axis. The higher the absolute value of an ASV’s score along the x-axis, the more strongly the ASV shifts a prediction of a sample towards a delta. Positive SHAP values push the prediction towards the Athabasca River Delta; negative SHAP values push prediction towards the Peace River.


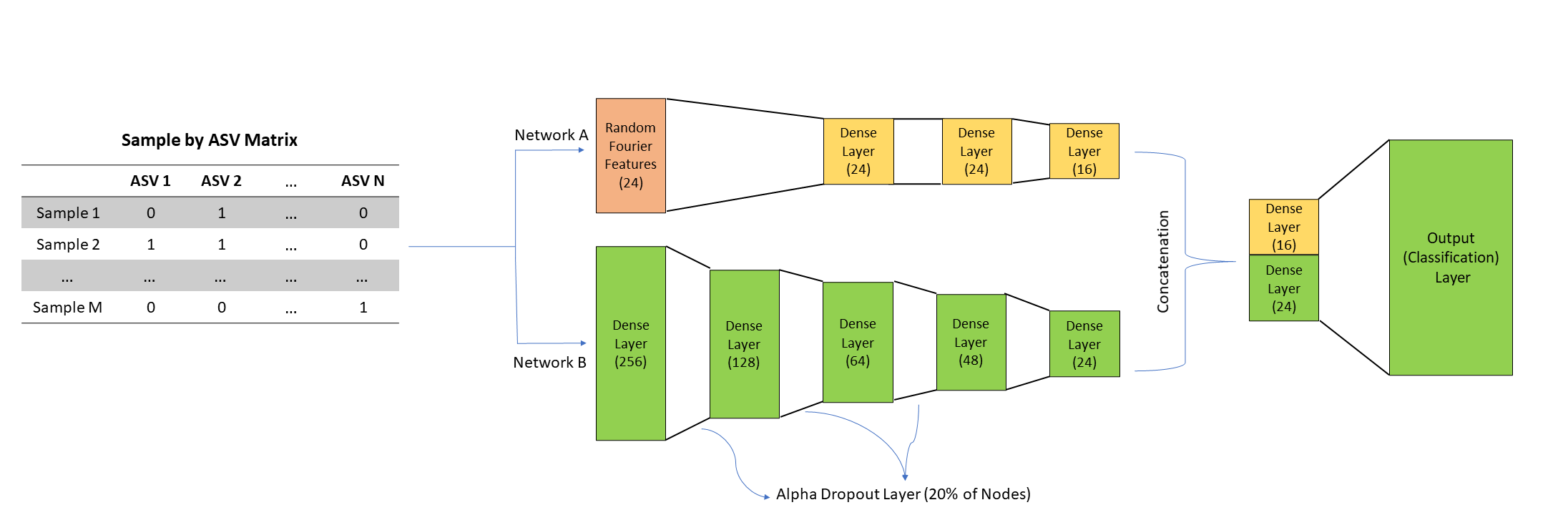


Suppl Figure 12: An overview of the neural network used by LANDMark. Input data, the sample by ASV matrix, is fed into two different networks, Network A and Network B. A Random Fourier Features transformation is applied to the input data, projecting it into a 24-Dimensional space. This information is then passed into the dense layers of Network A. The input data is directly used by Network B and the information from the network is compressed as it passes through the network into the deeper layers. The output layer uses the representations learned by the last layers Network A and B to arrive at a classification. The full network learns to classify the training data by adjusting the weights in each of the dense layers via backpropagation. The dropout layers work by randomly re-setting a fraction of the neurons at each epoch. This regularizes the network and helps prevent overfitting.

Suppl Figure 13: Analysis of how the number of estimators (left) and number of features considered (right) impact the performance of Random Forest, Extra Trees, LANDMark (Oracle), and LANDMark (No Oracle) classifiers. Individual LANDMark trees. The 95% confidence interval around each set of results for each estimator is drawn.


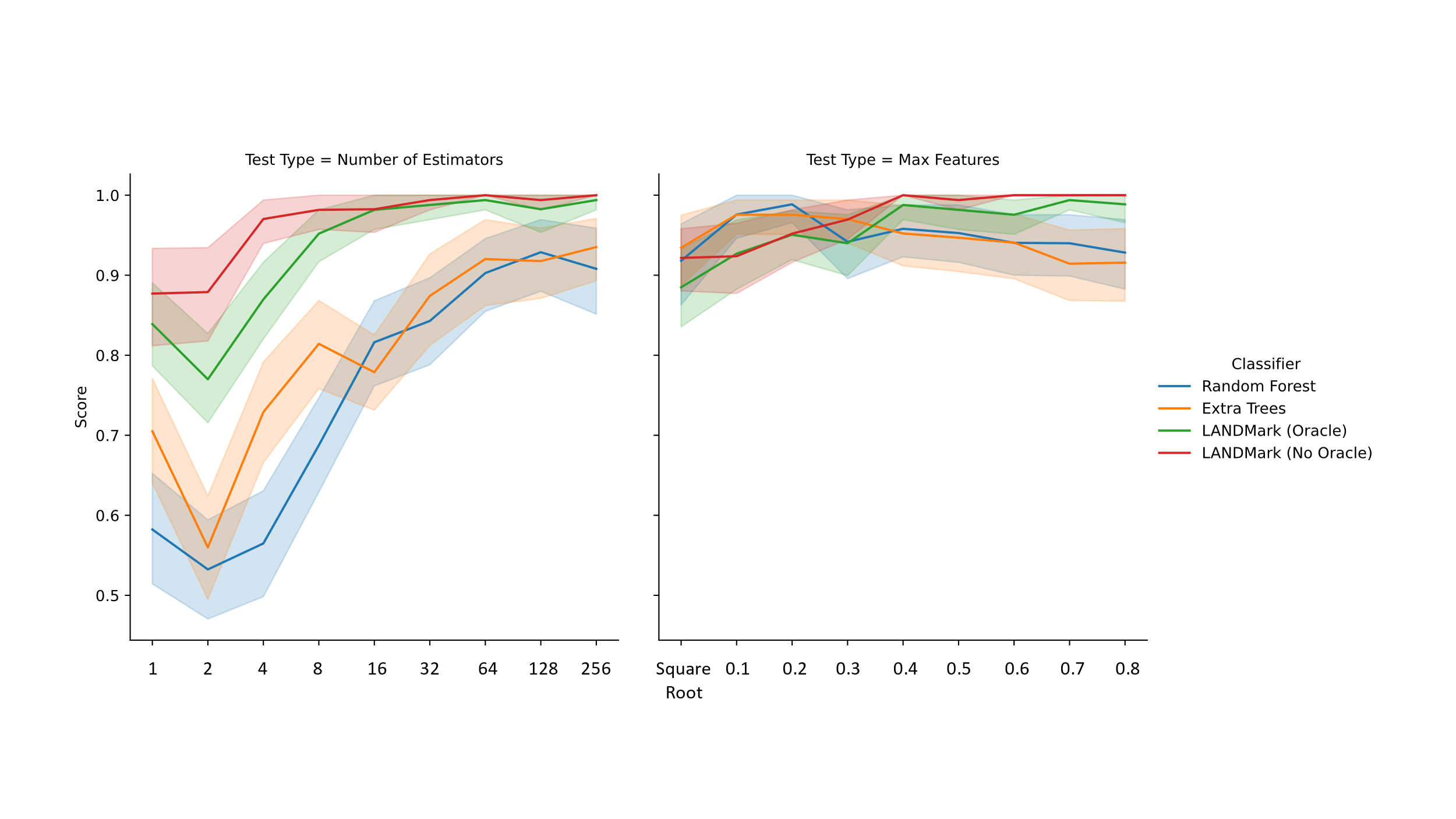

Supplement: Supplementary file 4 — Additional file 4. Figures 1 to 12. [file 12859_2022_4631_MOESM4_ESM.docx]
