# Supplementary material for: LANDMark: an ensemble approach to the supervised selection of biomarkers in high-throughput sequencing data
Source: BMC Bioinformatics. 2022 Mar 31;23:110. doi: 10.1186/s12859-022-04631-z (PMC8969335; doi:10.1186/s12859-022-04631-z)
Supplement: Supplementary file 5 — Additional file 5. Algorithm One. [file 12859_2022_4631_MOESM5_ESM.docx]

**Algorithm One: Pseudo-code of the LANDMark Algorithm**

***LANDMarkClassifier(N, n, m, c, o).fit(X, y)***

*Inputs:*

X – The training samples

y – The training labels

N – The number of trees

n – The maximum number of features to consider

m – The minimum number of samples upon which to split

c – The maximum depth of each tree

o – Whether to use a random linear oracle as the initial split function

*Output:*

A tree ensemble*,* $T= \left\{ t_{1},\ldots t_{N} \right\}$

1. For I = 1 to N
   1. Create the root note of the tree: $t_{i}=Node(N, n, m,c, o, c_{current}=1)$
   2. Split the training data and return the final tree: $t_{i}.GetSplit(X, y)$
   3. Sum the feature importance scores and normalize to a sum of 1
2. Sum the feature importance scores for each tree and normalize to a sum of 1

***GetSplit(X, y)***

*Inputs:*

X – The training samples

y – The training labels

*Output:*

A fully grown tree*,* $t$

1. Determine the probability,$p_{sample}$, of a sample reading the node
2. Determine the probability of each class, $p_{class}$ label in y
3. Determine which hypersurface will be randomly selected, H
4. If $\left| X \right|\leq m or Entropy\left| y \right|==0 or c_{current}\geq c$:
   1. Create a leaf such that the label of this leaf corresponds to the class with the maximum probability, $max(p_{class})$
   2. Return the leaf
5. If $o== True and c_{current}==1$:
   1. Without replacement, select two unique samples from X at random
   2. Calculate the midpoint between the two samples
   3. Save the normal vector, V, and the intercept, I,
   4. $X_{L}$ and $y_{L}$ are all the samples above the hyperplane defined by V and I
   5. $X_{R}$ and $y_{R}$ are all the samples at or below the hyperplane defined by V and I
   6. Create a new node $Node\left( N, n, m,c, o, c_{current}=c_{current}+1 \right).GetSplit(X_{L}, y_{L})$
   7. Create a new node $Node\left( N, n, m,c, o, c_{current}=c_{current}+1 \right).GetSplit(X_{R}, y_{R})$
6. If $c_{current}>1$:
   1. For each linear model^†^ trained using L2 regularization:
      1. If the dimensionality of $X \geq4$ Randomly select *n* features
      2. Create a bootstrapped sample of the training data, $X_{boot}$ and $y_{boot}$
      3. Train on the bootstrapped data and selected features and return the model
   2. For each linear model^†^ trained using L1 regularization:
      1. Create a bootstrapped sample of the training data, $X_{boot}$ and $y_{boot}$
      2. Train on the bootstrapped data and return the model
   3. If $\left| X \right|\geq32$:
      1. If the dimensionality of $X \geq4$ Randomly select *n* features
      2. Train a neural network model^†^ and return the model
   4. Calculate the information gain for each model’s split using *X*
   5. For each model $X_{L}$ and $y_{L}$ are all the samples above the hypersurface defined by the model and *H* while $X_{R}$ and $y_{R}$ are all the samples at or below the hypersurface defined by the model and *H*
   6. Find the set of models with the maximum information gain and select a model at random from this set
   7. Calculate the Shapley scores / weights for each feature and weight these scores by $p_{sample}$ and the best information gain ^♦^
   8. Set the current node’s splitting function to the best model and save the feature importance scores
   9. Create a new node $Node\left( N, n, m,c, o, c_{current}=c_{current}+1 \right).GetSplit(X_{L}, y_{L})$
   10. Create a new node $Node\left( N, n, m,c, o, c_{current}=c_{current}+1 \right).GetSplit(X_{R}, y_{R})$

*† - The models used by LANDMark and hyperparameter settings for these models can be found Table 6 and the architecture of the neural network model is described in the manuscript.*

*♦ - For linear models we sum the absolute value of all coefficients. The summed scores are then transformed into relative scores such that they sum to one. For the neural network model, the Shapley scores are calculated using the ‘GradientExplainer’ function from the ‘shap’ package. The mean of the absolute value of the results are then used as proxy for feature importance.*
